# Mapping spaces and automorphism groups of toric noncommutative spaces

**DOI:** 10.1007/s11005-017-0957-8

**Published:** 2017-04-07

**Authors:** Gwendolyn E. Barnes, Alexander Schenkel, Richard J. Szabo

**Affiliations:** 1 Department of Mathematics, Heriot-Watt University, Edinburgh, EH14 4AS UK; 2 Maxwell Institute for Mathematical Sciences, Edinburgh, UK; 3 The Higgs Centre for Theoretical Physics, Edinburgh, UK; 4 Fakultät für Mathematik, Universität Regensburg, 93040 Regensburg, Germany; 5 School of Mathematical Sciences, University of Nottingham, University Park, Nottingham, NG7 2RD UK

**Keywords:** Noncommutative geometry, Torus actions, Sheaves, Exponential objects, Automorphism groups, 16T05, 18F20, 53D55, 81R60

## Abstract

We develop a sheaf theory approach to toric noncommutative geometry which allows us to formalize the concept of mapping spaces between two toric noncommutative spaces. As an application, we study the ‘internalized’ automorphism group of a toric noncommutative space and show that its Lie algebra has an elementary description in terms of braided derivations.

## Introduction and summary

Toric noncommutative spaces are among the most studied and best understood examples in noncommutative geometry. Their function algebras *A* carry a coaction of a torus Hopf algebra *H*, whose cotriangular structure dictates the commutation relations in *A*. Famous examples are given by the noncommutative tori [27], the Connes–Landi spheres [15] and related deformed spaces [14]. More broadly, toric noncommutative spaces can be regarded as special examples of noncommutative spaces that are obtained by Drinfeld twist (or 2-cocycle) deformations of algebras carrying a Hopf algebra (co)action, see, e.g., [4–6] and references therein. For an algebraic geometry perspective on toric noncommutative varieties, see [12].

Noncommutative differential geometry on toric noncommutative spaces, and more generally on noncommutative spaces obtained by Drinfeld twist deformations, is far developed and well understood. Vector bundles (i.e., bimodules over *A*) have been studied in [4], where also a theory of noncommutative connections on bimodules was developed. These results were later formalized within the powerful framework of closed braided monoidal categories and therefore generalized to certain nonassociative spaces (obtained by cochain twist deformations) in [5, 6]. Examples of noncommutative principal bundles (i.e., Hopf–Galois extensions [9, 10]) in this framework were studied in [20], and these constructions were subsequently abstracted and generalized in [1]. In applications to noncommutative gauge theory, moduli spaces of instantons on toric noncommutative spaces were analyzed in [7, 8, 11, 13], while analogous moduli spaces of self-dual strings in higher noncommutative gauge theory were considered by [25].

Despite all this recent progress in understanding the geometry of toric noncommutative spaces, there is one very essential concept missing: Given two toric noncommutative spaces, say *X* and *Y*, we would like to have a ‘space of maps’ $Y^X$ from *X* to *Y*. The problem with such mapping spaces is that they will in general be ‘infinite-dimensional,’ just like the space of maps between two finite-dimensional manifolds is generically an infinite-dimensional manifold. In this paper, we propose a framework where such ‘infinite-dimensional’ toric noncommutative spaces may be formalized and which in particular allows us to describe the space of maps between any two toric noncommutative spaces. Our approach makes use of sheaf theory: Denoting by ${^{H}}\mathscr {S}$ the category of toric noncommutative spaces, we show that there is a natural site structure on ${^{H}}\mathscr {S}$ which generalizes the well-known Zariski site of algebraic geometry to the toric noncommutative setting. The category of generalized toric noncommutative spaces is then given by the sheaf topos ${^{H}}\mathscr {G}:= \mathrm {Sh}({^{H}}\mathscr {S})$, and we show that there is a fully faithful embedding ${^{H}}\mathscr {S}\rightarrow {^{H}}\mathscr {G}$ which allows us to equivalently regard toric noncommutative spaces as living in this bigger category. The advantage of the bigger category ${^{H}}\mathscr {G}$ is that it enjoys very good categorical properties; in particular, it admits all exponential objects. We can therefore make sense of the ‘space of maps’ $Y^X$ as a generalized toric noncommutative space in ${^{H}}\mathscr {G}$, i.e., as a sheaf on the site ${^{H}}\mathscr {S}$. As an application, we study the ‘internalized’ automorphism group $\mathrm {Aut}(X)$ of a toric noncommutative space *X*, which is a certain subobject in ${^{H}}\mathscr {G}$ of the self-mapping space $X^X$. Using synthetic geometry techniques, we are able to compute the Lie algebra of $\mathrm {Aut}(X)$ and we show that it can be identified with the braided derivations considered in [4, 6]. Hence, our concept of automorphism groups ‘integrates’ braided derivations to finite (internalized) automorphisms, which is an open problem in toric noncommutative geometry that cannot be solved by more elementary techniques.

Besides giving rise to a very rich concept of ‘internalized’ automorphism groups of toric noncommutative spaces, there are many other applications and problems which can be addressed with our sheaf theory approach to toric noncommutative geometry. For example, the mapping spaces $Y^X$ may be used to describe the spaces of field configurations for noncommutative sigma models, see, e.g., [16–18, 24]. Due to the fact that the mapping space $Y^X$ captures many more maps than the *set* of morphisms $\mathrm {Hom}(X,Y)$ (compare with Example 5.3 in the main text), this will lead to a much richer structure of noncommutative sigma models than those discussed previously. Another immediate application is to noncommutative principal bundles: It was observed in [10] that the definition of a good notion of gauge transformations for noncommutative Hopf–Galois extensions is somewhat problematic, because there are in general not enough algebra automorphisms of the total space algebra. To the best of our knowledge, this problem has not yet been solved. Using our novel sheaf theory techniques, we can give a natural definition of an ‘internalized’ gauge group for toric noncommutative principal bundles $P\rightarrow X$ by carving out a subobject in ${^{H}}\mathscr {G}$ of the ‘internalized’ automorphism group $\mathrm {Aut}(P)$ of the total space which consists of all maps that preserve the structure group action and the base space.

The outline of the remainder of this paper is as follows: In Sect. 2, we recall some preliminary results concerning cotriangular torus Hopf algebras *H* and their comodules, which form symmetric monoidal categories ${^{H}}\mathscr {M}$. In Sect. 3, we study algebra objects in ${^{H}}\mathscr {M}$ whose commutation relations are controlled by the cotriangular structure on *H*. We establish a category of finitely presented algebra objects ${^{H}}\mathscr {A}_{\mathrm {fp}}$, which contains noncommutative tori, Connes–Landi spheres and related examples, and study its categorical properties, including coproducts, pushouts and localizations. The category of toric noncommutative spaces ${^{H}}\mathscr {S}$ is then given by the opposite category of ${^{H}}\mathscr {A}_{\mathrm {fp}}$, and we show in Sect. 4 that ${^{H}}\mathscr {S}$ can be equipped with the structure of a site. In Sect. 5, we introduce and study the sheaf topos ${^{H}}\mathscr {G}$ whose objects are sheaves on ${^{H}}\mathscr {S}$ which we interpret as generalized toric noncommutative spaces. We show that the Yoneda embedding factorizes through ${^{H}}\mathscr {G}$ (i.e., that our site is subcanonical) and hence obtain a fully faithful embedding ${^{H}}\mathscr {S}\rightarrow {^{H}}\mathscr {G}$ of toric noncommutative spaces into generalized toric noncommutative spaces. An explicit description of the exponential objects $Y^X$ in ${^{H}}\mathscr {G}$ is given, which in particular allows us to formalize and study the mapping space between two toric noncommutative spaces. Using a simple example, it is shown in which sense the mapping spaces $Y^X$ are richer than the morphism sets $\mathrm {Hom}(X,Y)$ (cf. Example 5.3). In Sect. 6, we apply these techniques to define an ‘internalized’ automorphism group $\mathrm {Aut}(X)$ of a toric noncommutative space *X*, which arises as a certain subobject in ${^{H}}\mathscr {G}$ of the self-mapping space $X^X$. It is important to stress that $\mathrm {Aut}(X)$ is in general not representable, i.e., it has no elementary description in terms of a Hopf algebra, and hence, it is a truly generalized toric noncommutative space described by a sheaf on ${^{H}}\mathscr {S}$. The Lie algebra of $\mathrm {Aut}(X)$ is computed in Sect. 7 by using techniques from synthetic (differential) geometry [19, 21, 26]. We then show in Sect. 8 that the Lie algebra of $\mathrm {Aut}(X)$ can be identified with the braided derivations of the function algebra of *X*. Hence, in contrast to $\mathrm {Aut}(X)$, its Lie algebra of infinitesimal automorphisms has an elementary description. This identification is rather technical, and it relies on a fully faithful embedding ${^{H}}\mathscr {M}_{\mathrm {dec}}\rightarrow \mathrm {Mod}_{\underline{K}}({^{H}}\mathscr {G})$ of a certain full subcategory (called decomposables) of the category of left *H*-comodules ${^{H}}\mathscr {M}$ into the category of $\underline{K}$-module objects in the sheaf topos ${^{H}}\mathscr {G}$, where $\underline{K}$ denotes the line object in this topos; the technical details are presented in “Appendix.”

## Hopf algebra preliminaries

In this paper, all vector spaces will be over a fixed field $\mathbb {K}$ and the tensor product of vector spaces will be denoted simply by $\otimes$.

The Hopf algebra $H:= \mathcal {O}(\mathbb {T}^n)$ of functions on the algebraic *n*-torus $\mathbb {T}^n$ is defined as follows: As a vector space, *H* is spanned by the basis

2.1 $$\begin{aligned} \big \{t_{{\varvec{m}}}{:}\,{\varvec{m}} = (m_1,\ldots ,m_n)\in \mathbb {Z}^{n}\big \}, \end{aligned}$$

on which we define a (commutative and associative) product and unit by

2.2 $$\begin{aligned} t_{{\varvec{m}}}\,t_{{\varvec{m^\prime }}} = t_{{\varvec{m}} + {\varvec{m^\prime }}},\quad \mathbbm {1}_H^{} = t_{{\varvec{0}}}. \end{aligned}$$

The (cocommutative and coassociative) coproduct, counit and antipode in *H* are given by

2.3 $$\begin{aligned} \Delta (t_{{\varvec{m}}}) = t_{{\varvec{m}}}\otimes t_{{\varvec{m}}},\quad \epsilon (t_{{\varvec{m}}}) =1,\quad S(t_{{\varvec{m}}}) = t_{-{\varvec{m}}}. \end{aligned}$$

We choose a cotriangular structure on *H*, i.e., a linear map $R{:}\,H\otimes H\rightarrow \mathbb {K}$ satisfying

2.4a $$\begin{aligned} R(f\,g \otimes h)&= R(f\otimes h_{(1)}) ~R(g\otimes h_{(2)}), \end{aligned}$$

2.4b $$\begin{aligned} R(f\otimes g\,h)&= R(f_{(1)} \otimes h)~R(f_{(2)}\otimes g),\end{aligned}$$

2.4c $$\begin{aligned} \epsilon (h)\,\epsilon (g)&=R\left( h_{(1)}\otimes g_{(1)}\right) ~R\left( g_{(2)}\otimes h_{(2)}\right) , \end{aligned}$$

for all $f,g,h\in H$, where we have used Sweedler notation $\Delta (h) = h_{(1)}\otimes h_{(2)}$ (with summation understood) for the coproduct in *H*. The quasi-commutativity condition $g_{(1)}\,h_{(1)} \,R(h_{(2)}\otimes g_{(2)}) = R(h_{(1)}\otimes g_{(1)})\, h_{(2)}\, g_{(2)}$, for all $g,h\in H$, is automatically fulfilled because *H* is commutative and cocommutative. For example, if $\mathbb {K}=\mathbb {C}$ is the field of complex numbers, we may take the usual cotriangular structure defined by

2.5 $$\begin{aligned} R\left( t_{{\varvec{m}}}\otimes t_{{\varvec{m^\prime }}}\right) = \exp \left( \mathrm{i}\,\sum _{j,k=1}^n m_j \,\Theta ^{jk}\, m^\prime _{k}\right) , \end{aligned}$$

where $\Theta$ is an antisymmetric real $n{\times } n$-matrix, which plays the role of deformation parameters for the theory.

Let us denote by ${^{H}}\mathscr {M}$ the category of left *H*-comodules. An object in ${^{H}}\mathscr {M}$ is a pair $(V,\rho ^V)$, where *V* is a vector space and $\rho ^{V}{:}\,V\rightarrow H\otimes V$ is a left *H*-coaction on *V*, i.e., a linear map satisfying

2.6 $$\begin{aligned} (\mathrm {id}_H\otimes \rho ^V)\circ \rho ^V = (\Delta \otimes \mathrm {id}_V)\circ \rho ^V,\quad (\epsilon \otimes \mathrm {id}_V)\circ \rho ^V =\mathrm {id}_V. \end{aligned}$$

We follow the usual abuse of notation and denote objects $(V,\rho ^V)$ in ${^{H}}\mathscr {M}$ simply by *V* without displaying the coaction explicitly. We further use a Sweedler-like notation $\rho ^V(v) = v_{(-1)}\otimes v_{(0)}$ (with summation understood) for the left *H*-coactions. Then, (2.6) reads as

2.7 $$\begin{aligned} v_{(-1)} \otimes {v_{(0)}}_{(-1)}\otimes {v_{(0)}}_{(0)} = {v_{(-1)}}_{(1)} \otimes {v_{(-1)}}_{(2)}\otimes v_{(0)},\quad \epsilon (v_{(-1)}) \, v_{(0)} = v. \end{aligned}$$

A morphism $L{:}\,V\rightarrow W$ in ${^{H}}\mathscr {M}$ is a linear map preserving the left *H*-coactions, i.e.,

2.8a $$\begin{aligned} (\mathrm {id}_H\otimes L)\circ \rho ^{V} = \rho ^W\circ L, \end{aligned}$$

or in the Sweedler-like notation

2.8b $$\begin{aligned} v_{(-1)}\otimes L(v_{(0)}) =L(v)_{(-1)} \otimes L(v)_{(0)}, \end{aligned}$$

for all $$v\in V$$.

The category ${^{H}}\mathscr {M}$ is a monoidal category with tensor product of two objects *V* and *W* given by the tensor product $V\otimes W$ of vector spaces equipped with the left *H*-coaction

2.9 $$\begin{aligned} \rho ^{V\otimes W}{:}\,V\otimes W \longrightarrow H\otimes V\otimes W,~~v\otimes w \longmapsto v_{(-1)}\,w_{(-1)}\otimes v_{(0)}\otimes w_{(0)}. \end{aligned}$$

The monoidal unit in ${^{H}}\mathscr {M}$ is given by the one-dimensional vector space $\mathbb {K}$ with trivial left *H*-coaction $\mathbb {K}\rightarrow H\otimes \mathbb {K},~c\mapsto \mathbbm {1}_H^{}\otimes c$. The monoidal category ${^{H}}\mathscr {M}$ is symmetric with commutativity constraint

2.10 $$\begin{aligned} \tau _{V,W}^{}{:}\,V\otimes W \longrightarrow W\otimes V,~~ v\otimes w \longmapsto R(w_{(-1)}\otimes v_{(-1)})\, w_{(0)}\otimes v_{(0)}, \end{aligned}$$

for any two objects *V* and *W* in ${^{H}}\mathscr {M}$.

## Algebra objects

We are interested in spaces whose algebras of functions are described by certain algebra objects in the symmetric monoidal category ${^{H}}\mathscr {M}$. An algebra object in ${^{H}}\mathscr {M}$ is an object *A* in ${^{H}}\mathscr {M}$ together with two ${^{H}}\mathscr {M}$-morphisms $\mu _{A}^{}{:}\,A\otimes A\rightarrow A$ (product) and $\eta _{A}^{}{:}\,\mathbb {K}\rightarrow A$ (unit) such that the diagrams3.1in ${^{H}}\mathscr {M}$ commute. Because ${^{H}}\mathscr {M}$ is symmetric, we may additionally demand that the product $\mu _A^{}$ is compatible with the commutativity constraints in ${^{H}}\mathscr {M}$, i.e., the diagram3.2in ${^{H}}\mathscr {M}$ commutes. This amounts to demanding the commutation relations

3.3 $$\begin{aligned} a\,a^\prime = R\left( a^\prime _{(-1)}\otimes a_{(-1)}\right) ~a^\prime _{(0)}\,a_{(0)}, \end{aligned}$$

for all $a,a^\prime \in A$, where we have abbreviated the product by $\mu _A^{}(a\otimes a^\prime ) = a\,a^\prime$; in the following, we shall also use the compact notation $\mathbbm {1} _A^{}:= \eta _A^{}(1)\in A$ for the unit element in *A*, or sometimes just $\mathbbm {1}$. Such algebras are *not* commutative in the ordinary sense once we choose a nontrivial cotriangular structure as, for example, in (2.5), see also Example 3.5.

Let us introduce the category of algebras of interest.

### Definition 3.1

The category ${^{H}}\mathscr {A}$ has as objects all algebra objects in ${^{H}}\mathscr {M}$ which satisfy the commutativity constraint (3.2). The morphisms between two objects are all ${^{H}}\mathscr {M}$-morphisms $\kappa {:}\,A\rightarrow B$ which preserve products and units, i.e., for which $\mu _B^{}\circ \kappa \otimes \kappa = \kappa \circ \mu _A^{}$ and $\kappa \circ \eta _A^{} = \eta _B^{}$.

There is the forgetful functor $\mathrm {Forget}{:}\,{^{H}}\mathscr {A}\rightarrow {^{H}}\mathscr {M}$ which assigns to any object in ${^{H}}\mathscr {A}$ its underlying left *H*-comodule, i.e., $(A,\mu _A^{},\eta _A^{}) \mapsto A$. This functor has a left adjoint $\mathrm {Free}{:}\,{^{H}}\mathscr {M}\rightarrow {^{H}}\mathscr {A}$ which describes the free ${^{H}}\mathscr {A}$-algebra construction: Given any object *V* in ${^{H}}\mathscr {M}$, we consider the vector space

3.4 $$\begin{aligned} \mathcal {T}V := \bigoplus _{n\ge 0} \, V^{\otimes n}, \end{aligned}$$

with the convention $V^{\otimes 0} :=\mathbb {K}$. Then, $\mathcal {T}V$ is a left *H*-comodule when equipped with the coaction $\rho ^{\mathcal {T}V}{:}\,\mathcal {T}V \rightarrow H\otimes \mathcal {T}V$ specified by

3.5 $$\begin{aligned} \rho ^{\mathcal {T}V}\big (v_1\otimes \cdots \otimes v_n\big ) = {v_{1}}_{(-1)} \ldots {v_{n}}_{(-1)} \otimes {v_{1}}_{(0)}\otimes \cdots \otimes {v_{n}}_{(0)}. \end{aligned}$$

Moreover, $\mathcal {T}V$ is an algebra object in ${^{H}}\mathscr {M}$ when equipped with the product $\mu _{\mathcal {T}V}^{}{:}\,\mathcal {T}V\otimes \mathcal {T}V \rightarrow \mathcal {T}V$ specified by

3.6 $$\begin{aligned} \mu _{\mathcal {T}V}^{}\big ((v_1\otimes \cdots \otimes v_n)\otimes (v_{n+1}\otimes \cdots \otimes v_{n+m})\big )= v_1\otimes \cdots \otimes v_{n+m} \end{aligned}$$

and the unit $\eta _{\mathcal {T}V}^{}{:}\,\mathbb {K}\rightarrow \mathcal {T}V$ given by

3.7 $$\begin{aligned} \eta _{\mathcal {T}V}^{}(c)= c \in V^{\otimes 0} \subseteq \mathcal {T}V. \end{aligned}$$

The algebra object $\mathcal {T}V$ does not satisfy the commutativity constraint (3.2); hence, it is not an object of the category ${^{H}}\mathscr {A}$. We may enforce the commutativity constraint by taking the quotient of $\mathcal {T}V$ by the two-sided ideal $I\subseteq \mathcal {T}V$ generated by

3.8 $$\begin{aligned} v \otimes v^\prime - R\left( v^\prime _{(-1)}\otimes v_{(-1)}\right) ~v^\prime _{(0)}\otimes v_{(0)}, \end{aligned}$$

for all $v,v^\prime \in V$. The ideal *I* is stable under the left *H*-coaction, i.e., $\rho ^{\mathcal {T}V}{:}\,I \rightarrow H\otimes I$. Hence, the quotient

3.9 $$\begin{aligned} \mathrm {Free}(V) := \mathcal {T}V{/}I \end{aligned}$$

is an object in ${^{H}}\mathscr {A}$ when equipped with the induced left *H*-coaction, product and unit. Given now any ${^{H}}\mathscr {M}$-morphism $L{:}\,V\rightarrow W$, we define an ${^{H}}\mathscr {A}$-morphism $\mathrm {Free}(L){:}\,\mathrm {Free}(V)\rightarrow \mathrm {Free}(W)$ by setting

3.10 $$\begin{aligned} \mathrm {Free}(L) \big (v_1\otimes \cdots \otimes v_n\big ) = L(v_1)\otimes \cdots \otimes L(v_n). \end{aligned}$$

This is compatible with the quotients because of (2.8). Finally, let us confirm that $\mathrm {Free}{:}\,{^{H}}\mathscr {M}\rightarrow {^{H}}\mathscr {A}$ is the left adjoint of the forgetful functor $\mathrm {Forget}{:}\,{^{H}}\mathscr {A}\rightarrow {^{H}}\mathscr {M}$, i.e., that there exists a (natural) bijection

3.11 $$\begin{aligned} \mathrm {Hom}_{{^{H}}\mathscr {A}}^{}\big (\mathrm {Free}(V), A \big ) \simeq \mathrm {Hom}_{{^{H}}\mathscr {M}}^{}\big (V,\mathrm {Forget}(A)\big ) \end{aligned}$$

between the morphism sets, for any object *V* in ${^{H}}\mathscr {M}$ and any object *A* in ${^{H}}\mathscr {A}$. This is easy to see from the fact that any ${^{H}}\mathscr {A}$-morphism $\kappa {:}\,\mathrm {Free}(V)\rightarrow A$ is uniquely specified by its restriction to the vector space $V = V^{\otimes 1}\subseteq \mathrm {Free}(V)$ of generators and hence by an ${^{H}}\mathscr {M}$-morphism $V\rightarrow \mathrm {Forget}(A)$.

From a geometric perspective, the free ${^{H}}\mathscr {A}$-algebras $\mathrm {Free}(V)$ describe the function algebras on toric noncommutative planes. In order to capture a larger class of toric noncommutative spaces, we introduce a suitable concept of ideals for ${^{H}}\mathscr {A}$-algebras.

### Definition 3.2

Let *A* be an object in ${^{H}}\mathscr {A}$. An ${^{H}}\mathscr {A}$-ideal *I* of *A* is a two-sided ideal $I\subseteq A$ of the algebra underlying *A* which is stable under the left *H*-coaction, i.e., the coaction $\rho ^A$ induces a linear map $\rho ^A{:}\,I \rightarrow H\otimes I$.

This definition immediately implies

### Lemma 3.3

If *A* is an object in ${^{H}}\mathscr {A}$ and *I* is an ${^{H}}\mathscr {A}$-ideal of *A*, the quotient *A* / *I* is an object in ${^{H}}\mathscr {A}$ when equipped with the induced coaction, product and unit.

This lemma allows us to construct a variety of ${^{H}}\mathscr {A}$-algebras by taking quotients of free ${^{H}}\mathscr {A}$-algebras by suitable ${^{H}}\mathscr {A}$-ideals. We are particularly interested in the case where the object *V* in ${^{H}}\mathscr {M}$ that underlies the free ${^{H}}\mathscr {A}$-algebra $\mathrm {Free}(V)$ is finite-dimensional; geometrically, this corresponds to a finite-dimensional toric noncommutative plane. We shall introduce a convenient notation for this case: First, notice that the one-dimensional left *H*-comodules over the torus Hopf algebra $H = \mathcal {O}(\mathbb {T}^n)$ can be characterized by a label ${\varvec{m}}\in \mathbb {Z}^n$. The corresponding left *H*-coactions are given by

3.12 $$\begin{aligned} \rho ^{{\varvec{m}}}{:}\,\mathbb {K}\longrightarrow H\otimes \mathbb {K},~~c\longmapsto t_{{\varvec{m}}}\otimes c. \end{aligned}$$

We shall use the notation $\mathbb {K}_{{\varvec{m}}} := (\mathbb {K},\rho ^{{\varvec{m}}})$ for these objects in ${^{H}}\mathscr {M}$. The coproduct $\mathbb {K}_{{\varvec{m}}}\sqcup \mathbb {K}_{{\varvec{m^\prime }}}$ of two such objects is given by the vector space $\mathbb {K}\oplus \mathbb {K}\simeq \mathbb {K}^2$ together with the component-wise coaction, i.e.,

3.13 $$\begin{aligned} \rho ^{\mathbb {K}_{{\varvec{m}}}\sqcup \mathbb {K}_{{\varvec{m^\prime }}}} \big (c\oplus 0\big ) = t_{{\varvec{m}}}\otimes (c\oplus 0),\quad \rho ^{\mathbb {K}_{{\varvec{m}}}\sqcup \mathbb {K}_{{\varvec{m^\prime }}}} \big (0\oplus c\big ) = t_{{\varvec{m^\prime }}}\otimes (0\oplus c). \end{aligned}$$

The free ${^{H}}\mathscr {A}$-algebra corresponding to a finite coproduct of objects $\mathbb {K}_{{\varvec{m}}_i}$, for $i=1,\ldots ,N$, in ${^{H}}\mathscr {M}$ will be used frequently in this paper. Hence, we introduce the compact notation

3.14 $$\begin{aligned} F_{{\varvec{m}}_1,\ldots , {\varvec{m}}_N} := \mathrm {Free}\big (\mathbb {K}_{{\varvec{m}}_1}\sqcup \cdots \sqcup \mathbb {K}_{{\varvec{m}}_N}\big ). \end{aligned}$$

By construction, the ${^{H}}\mathscr {A}$-algebras $F_{{\varvec{m}}_1,\ldots , {\varvec{m}}_N}$ are generated by *N* elements $x_i\in F_{{\varvec{m}}_1,\ldots ,{\varvec{m}}_N}$ whose transformation property under the left *H*-coaction is given by $\rho ^{F_{{\varvec{m}}_1,\ldots , {\varvec{m}}_N}}(x_i) = t_{{\varvec{m}}_i}\otimes x_i$ and whose commutation relations read as

3.15 $$\begin{aligned} x_i \,x_j = R\left( t_{{\varvec{m}}_j}\otimes t_{{\varvec{m}}_i}\right) ~x_j\,x_i, \end{aligned}$$

for all $$i,j=1,\ldots ,N$$.

We can now introduce the category of finitely presented ${^{H}}\mathscr {A}$-algebras.

### Definition 3.4

An object *A* in ${^{H}}\mathscr {A}$ is finitely presented if it is isomorphic to the quotient $F_{{\varvec{m}}_1,\ldots , {\varvec{m}}_N}/I$ of a free ${^{H}}\mathscr {A}$-algebra $F_{{\varvec{m}}_1,\ldots , {\varvec{m}}_N}$ by an ${^{H}}\mathscr {A}$-ideal $I= (f_k)$ that is generated by a finite number of elements $f_k\in F_{{\varvec{m}}_1,\ldots , {\varvec{m}}_N}$, for $k=1,\ldots , M$, with $\rho ^{F_{{\varvec{m}}_1,\ldots , {\varvec{m}}_N}}(f_k) = t_{{\varvec{n}}_k}\otimes f_k$, for some ${\varvec{n}}_k\in \mathbb {Z}^n$. We denote by ${^{H}}\mathscr {A}_{\mathrm {fp}}$ the full subcategory of ${^{H}}\mathscr {A}$ whose objects are all finitely presented ${^{H}}\mathscr {A}$-algebras.

### Example 3.5

Let us consider the case $\mathbb {K}=\mathbb {C}$ and *R* given by (2.5). Take the free ${^{H}}\mathscr {A}$-algebra generated by $x_i$ and $x_i^*$, for $i=1,\ldots ,N$, with left *H*-coaction specified by $x_i\mapsto t_{{\varvec{m}}_i}\otimes x_i$ and $x_i^*\mapsto t_{-{\varvec{m}}_i}\otimes x_i^*$, for some ${\varvec{m}}_i\in \mathbb {Z}^n$; in the notation above, we consider the free ${^{H}}\mathscr {A}$-algebra $F_{{\varvec{m}}_1,\ldots ,{\varvec{m}}_N,-{\varvec{m}}_1,\ldots ,-{\varvec{m}}_N}$ and denote the last *N* generators by $x_i^*:= x_{N+i}$, for $i=1,\ldots ,N$. The algebra of (the algebraic version of) the $2N{-}1$-dimensional Connes–Landi sphere is obtained by taking the quotient with respect to the ${^{H}}\mathscr {A}$-ideal

3.16 $$\begin{aligned} I_{\mathbb {S}_\Theta ^{2N-1}} := \left( {\sum \limits _{i=1}^N} \, x_i^*\,x_i -\mathbbm {1}\right) , \end{aligned}$$

which implements the unit sphere relation. The algebra of the *N*-dimensional noncommutative torus is obtained by taking the quotient with respect to the ${^{H}}\mathscr {A}$-ideal

3.17 $$\begin{aligned} I_{\mathbb {T}_\Theta ^N} := \big (x_i^*\,x_i -\mathbbm {1}{:}\,i=1,\ldots ,N\big ). \end{aligned}$$

To obtain also the even dimensional Connes–Landi spheres, we consider the free ${^{H}}\mathscr {A}$-algebra $F_{{\varvec{m}}_1,\ldots ,{\varvec{m}}_N,-{\varvec{m}}_1,\ldots ,-{\varvec{m}}_N,{\varvec{0}}}$, where the additional generator $x_{2N+1}$ has trivial *H*-coaction $x_{2N+1}\mapsto \mathbbm {1}_H\otimes x_{2N+1}$, and take the quotient with respect to the ${^{H}}\mathscr {A}$-ideal

3.18 $$\begin{aligned} I_{\mathbb {S}_\Theta ^{2N}} := \left( {\sum \limits _{i=1}^N} \, x_i^*\,x_i + (x_{2N+1})^2 -\mathbbm {1}\right) . \end{aligned}$$

All these examples are $*$-algebras with involution defined by $x_i \mapsto x_i^*$ and $x_{2N+1}^*= x_{2N+1}$. An example which is not a $*$-algebra is the free ${^{H}}\mathscr {A}$-algebra $F_{{\varvec{m}}}$, for some ${\varvec{m}}\ne {\varvec{0}}$ in $\mathbb {Z}^n$, which we may interpret as the algebra of (anti)holomorphic polynomials on $\mathbb {C}$.

We will now study some properties of the categories ${^{H}}\mathscr {A}$ and ${^{H}}\mathscr {A}_{\mathrm {fp}}$ that will be used in the following. First, let us notice that the category ${^{H}}\mathscr {A}$ has (finite) coproducts: Given two objects *A* and *B* in ${^{H}}\mathscr {A}$, their coproduct $A\sqcup B$ is the object in ${^{H}}\mathscr {A}$ whose underlying left *H*-comodule is $A\otimes B$ [with coaction $\rho ^{A\sqcup B} := \rho ^{A\otimes B}$ given in (2.9)] and whose product $\mu _{A\sqcup B}$ and unit $\eta _{A\sqcup B}$ are characterized by

3.19a $$\begin{aligned} (a\otimes b) \,(a^\prime \otimes b^\prime )&:= R\left( a_{(-1)}^\prime \otimes b_{(-1)}\right) ~(a\,a_{(0)}^\prime )\otimes (b_{(0)}\,b^\prime ),\end{aligned}$$

3.19b $$\begin{aligned} \mathbbm {1}_{A\sqcup B}&:= \mathbbm {1}_A^{}\otimes \mathbbm {1}_B^{}. \end{aligned}$$

The canonical inclusion ${^{H}}\mathscr {A}$-morphisms $\iota _1^{}{:}\,A\rightarrow A\sqcup B$ and $\iota _2^{}{:}\,B\rightarrow A\sqcup B$ are given by

3.20 $$\begin{aligned} \iota _{1}^{}(a) = a\otimes \mathbbm {1}_B^{},\quad \iota _2^{}(b) = \mathbbm {1}_A^{}\otimes b, \end{aligned}$$

for all $a\in A$ and $b\in B$. The coproduct $A\sqcup B$ of two finitely presented ${^{H}}\mathscr {A}$-algebras *A* and *B* is finitely presented: If $A = F_{{\varvec{m}}_1,\ldots , {\varvec{m}}_N}/(f_k)$ and $B = F_{{\varvec{m^\prime }}_1,\ldots , {\varvec{m^\prime }}_{N^\prime }}/(f^\prime _{k^\prime })$, then

3.21 $$\begin{aligned} A\sqcup B \simeq F_{{\varvec{m}}_1,\ldots , {\varvec{m}}_N,{\varvec{m^\prime }}_1,\ldots , {\varvec{m^\prime }}_{N^\prime }}{\big /}\big (f_k\otimes \mathbbm {1}, \, \mathbbm {1}\otimes f^\prime _{k^\prime }\big ), \end{aligned}$$

where we have identified $F_{{\varvec{m}}_1,\ldots , {\varvec{m}}_N,{\varvec{m^\prime }}_1,\ldots , {\varvec{m^\prime }}_{N^\prime }}\simeq F_{{\varvec{m}}_1,\ldots , {\varvec{m}}_N} \sqcup F_{{\varvec{m^\prime }}_1,\ldots , {\varvec{m^\prime }}_{N^\prime }}$. Consequently, the category ${^{H}}\mathscr {A}_{\mathrm {fp}}$ has finite coproducts.

In addition to coproducts, we also need pushouts in ${^{H}}\mathscr {A}$ and ${^{H}}\mathscr {A}_{\mathrm {fp}}$, which are given by colimits of the form3.22in ${^{H}}\mathscr {A}$ or ${^{H}}\mathscr {A}_{\mathrm {fp}}$. Such pushouts exist and can be constructed as follows: Consider first the case where we work in the category ${^{H}}\mathscr {A}$. We define

3.23 $$\begin{aligned} A\sqcup _C^{} B := A\sqcup B{/}I, \end{aligned}$$

where *I* is the ${^{H}}\mathscr {A}$-ideal generated by $\kappa (c)\otimes \mathbbm {1}_B^{} - \mathbbm {1}_{A}^{}\otimes \zeta (c)$, for all $c\in C$. The dashed ${^{H}}\mathscr {A}$-morphisms in (3.22) are given by

3.24 $$\begin{aligned} A\longrightarrow A\sqcup _C^{} B,~~a\longmapsto [a\otimes \mathbbm {1}_B^{}],\quad B\longrightarrow A\sqcup _C^{} B,~~b\longmapsto [\mathbbm {1}_{A}^{}\otimes b]. \end{aligned}$$

It is easy to confirm that $A\sqcup _C^{}B$ defined above is a pushout of the diagram (3.22). Moreover, the pushout of finitely presented ${^{H}}\mathscr {A}$-algebras is finitely presented: If $A = F_{{\varvec{m}}_1,\ldots , {\varvec{m}}_N}/(f_k)$, $B = F_{{\varvec{m^\prime }}_1,\ldots , {\varvec{m^\prime }}_{N^\prime }}/(f^\prime _{k^\prime })$ and $C = F_{{\varvec{m^{\prime \prime }}}_1,\ldots , {\varvec{m^{\prime \prime }}}_{N^{\prime \prime }}}/ (f^{\prime \prime }_{k^{\prime \prime }})$, then

3.25 $$\begin{aligned} A\sqcup _C^{} B \simeq F_{{\varvec{m}}_1,\ldots , {\varvec{m}}_N,{\varvec{m^\prime }}_1,\ldots , {\varvec{m^\prime }}_{N^\prime }}{\big /}\big (f_k\otimes \mathbbm {1},\, \mathbbm {1}\otimes f^\prime _{k^\prime }, \, \kappa (x^{\prime \prime }_{i})\otimes \mathbbm {1} - \mathbbm {1}\otimes \zeta (x^{\prime \prime }_i) \big ), \end{aligned}$$

where $x^{\prime \prime }_i$, for $i=1,\ldots , N^{\prime \prime }$, are the generators of *C*. The isomorphism in (3.25) follows from the fact that the quotient by the *finite* number of extra relations $\kappa (x^{\prime \prime }_{i})\otimes \mathbbm {1} - \mathbbm {1}\otimes \zeta (x^{\prime \prime }_i)$, for all generators $x^{\prime \prime }_i$ of *C*, is sufficient to describe the ${^{H}}\mathscr {A}$-ideal *I* in (3.23) for finitely presented ${^{H}}\mathscr {A}$-algebras *C*: We can recursively use the identities [valid on the right-hand side of (3.25)]

3.26a $$\begin{aligned} \big [\kappa (x^{\prime \prime }_i\,c)\otimes \mathbbm {1} - \mathbbm {1}\otimes \zeta (x^{\prime \prime }_i\,c)\big ]&= \big [\big (\kappa (x^{\prime \prime }_i)\otimes \mathbbm {1} \big ) \, \big (\kappa (c)\otimes \mathbbm {1} - \mathbbm {1} \otimes \zeta (c)\big )\big ], \end{aligned}$$

3.26b $$\begin{aligned} \big [\kappa (c\, x^{\prime \prime }_i)\otimes \mathbbm {1} - \mathbbm {1}\otimes \zeta (c\,x^{\prime \prime }_i)\big ]&= \big [\big (\kappa (c)\otimes \mathbbm {1} - \mathbbm {1}\otimes \zeta (c)\big )\, \big (\kappa (x^{\prime \prime }_i)\otimes \mathbbm {1} \big ) \big ], \end{aligned}$$

for all generators $x^{\prime \prime }_i$ and elements $c\in C$, in order to show that the ${^{H}}\mathscr {A}$-ideal *I* is equivalently generated by $\kappa (x^{\prime \prime }_{i})\otimes \mathbbm {1} - \mathbbm {1} \otimes \zeta (x^{\prime \prime }_i)$. Consequently, the category ${^{H}}\mathscr {A}_{\mathrm {fp}}$ has pushouts.

We also need the localization of ${^{H}}\mathscr {A}$-algebras *A* with respect to a single *H-coinvariant* element $s \in A$, i.e., $\rho ^A(s) = \mathbbm {1}_H^{}\otimes s$. Localization amounts to constructing an ${^{H}}\mathscr {A}$-algebra $A[s^{-1}]$ together with an ${^{H}}\mathscr {A}$-morphism $\ell _s{:}\,A\rightarrow A[s^{-1}]$ that maps the element $s\in A$ to an invertible element $\ell _s(s)\in A[s^{-1}]$ and that satisfies the following universal property: If $\kappa {:}\,A\rightarrow B$ is another ${^{H}}\mathscr {A}$-morphism such that $\kappa (s)\in B$ is invertible, then $\kappa$ factors though $\ell _s{:}\,A\rightarrow A[s^{-1}]$, i.e., there exists a unique ${^{H}}\mathscr {A}$-morphism $A[s^{-1}]\rightarrow B$ making the diagram3.27commute. We now show that the ${^{H}}\mathscr {A}$-algebra

3.28a $$\begin{aligned} A[s^{-1}] := A\sqcup F_{{\varvec{0}}}{/}\left( s\otimes x- \mathbbm {1}_{A\sqcup F_{{\varvec{0}}}}^{}\right) \end{aligned}$$

together with the ${^{H}}\mathscr {A}$-morphism

3.28b $$\begin{aligned} \ell _s{:}\,A\longrightarrow A[s^{-1}],~~a\longmapsto [a\otimes \mathbbm {1}_{F_{{\varvec{0}}}}^{}] \end{aligned}$$

is a localization of *A* with respect to the *H*-coinvariant element $s\in A$. The inverse of $\ell _s(s) = [s\otimes \mathbbm {1}_{F_{{\varvec{0}}}}^{}]$ exists, and it is given by the new generator $[\mathbbm {1}_A^{}\otimes x]\in A[s^{-1}]$; then, the inverse of $\ell _s(s^n)$ is $[\mathbbm {1}_A^{}\otimes x^n]$, because $[s\otimes \mathbbm {1}_{F_{{\varvec{0}}}}^{}]$ and $[\mathbbm {1}_A^{}\otimes x]$ commute in $A[s^{-1}]$, cf. (3.3), (3.19) and use the fact that *x* and *s* are coinvariants. Given now any ${^{H}}\mathscr {A}$-morphism $\kappa {:}\,A\rightarrow B$ such that $\kappa (s)\in B$ is invertible, say by $t\in B$, there is a unique ${^{H}}\mathscr {A}$-morphism $A[s^{-1}]\rightarrow B$ specified by $[a\otimes x^{n}]\mapsto \kappa (a)\, t^n$ that factors $\kappa$ through $\ell _s{:}\,A\rightarrow A[s^{-1}]$. Finally, the localization of finitely presented ${^{H}}\mathscr {A}$-algebras is finitely presented: If $A= F_{{\varvec{m}}_1,\ldots ,{\varvec{m}}_N}/(f_k)$, then

3.29 $$\begin{aligned} A[s^{-1}]\simeq F_{{\varvec{m}}_1,\ldots ,{\varvec{m}}_N,{\varvec{0}}}{/}\left( f_k\otimes \mathbbm {1} ,\, s\otimes x - \mathbbm {1}\right) . \end{aligned}$$

## Toric noncommutative spaces

From a geometric perspective, it is useful to interpret an object *A* in ${^{H}}\mathscr {A}_{\mathrm {fp}}$ as the ‘algebra of functions’ on a toric noncommutative space $X_A$. Similarly, a morphism $\kappa {:}\,A\rightarrow B$ in ${^{H}}\mathscr {A}_{\mathrm {fp}}$ is interpreted as the ‘pullback’ of a map $f{:}\,X_B\rightarrow X_A$ between toric noncommutative spaces, where due to contravariance of pullbacks the direction of the arrow is reversed when going from algebras to spaces. We shall use the more intuitive notation $\kappa = f^*{:}\,A\rightarrow B$ for the ${^{H}}\mathscr {A}_{\mathrm {fp}}$-morphism corresponding to $f{:}\,X_B\rightarrow X_A$. This can be made precise with

### Definition 4.1

The category of toric noncommutative spaces

4.1 $$\begin{aligned} {^{H}}\mathscr {S}:= \big ({^{H}}\mathscr {A}_{\mathrm {fp}}\big )^\mathrm {op}\end{aligned}$$

is the opposite of the category ${^{H}}\mathscr {A}_{\mathrm {fp}}$. Objects in ${^{H}}\mathscr {S}$ will be denoted by symbols like $X_A$, where *A* is an object in ${^{H}}\mathscr {A}_{\mathrm {fp}}$. Morphisms in ${^{H}}\mathscr {S}$ will be denoted by symbols like $f{:}\,X_{B}\rightarrow X_{A}$, and they are (by definition) in bijection with ${^{H}}\mathscr {A}_{\mathrm {fp}}$-morphisms $f^*{:}\,A\rightarrow B$.

As the category ${^{H}}\mathscr {A}_{\mathrm {fp}}$ has (finite) coproducts and pushouts, which we have denoted by $A\sqcup B$ and $A\sqcup _C^{} B$, its opposite category ${^{H}}\mathscr {S}$ has (finite) products and pullbacks. Given two objects $X_A$ and $X_B$ in ${^{H}}\mathscr {S}$, their product is given by

4.2a $$\begin{aligned} X_A\times X_B := X_{A\sqcup B}, \end{aligned}$$

together with the canonical projection ${^{H}}\mathscr {S}$-morphisms

4.2b $$\begin{aligned} \pi _{1}^{}{:}\,X_A\times X_B \longrightarrow X_A,\quad \pi _{2}^{}{:}\,X_A\times X_B \longrightarrow X_B \end{aligned}$$

specified by $\pi _{1}^*= \iota _{1}^{}{:}\,A\rightarrow A\sqcup B$ and $\pi _{2}^*= \iota _{2}^{}{:}\,B\rightarrow A\sqcup B$, where $\iota _{1}^{}$ and $\iota _{2}^{}$ are the canonical inclusion ${^{H}}\mathscr {A}_{\mathrm {fp}}$-morphisms for the coproduct in ${^{H}}\mathscr {A}_{\mathrm {fp}}$ [cf. (3.20)]. Pullbacks4.3in ${^{H}}\mathscr {S}$ are given by

4.4 $$\begin{aligned} X_A\times _{X_C}^{} X_B := X_{A\sqcup _C^{} B}, \end{aligned}$$

for $\kappa = f^*{:}\,C\rightarrow A$ and $\zeta = g^*{:}\,C\rightarrow B$ [cf. (3.22)]. The dashed arrows in (4.3) are specified by their corresponding ${^{H}}\mathscr {A}_{\mathrm {fp}}$-morphisms in (3.24).

We next introduce a suitable notion of covering for toric noncommutative spaces, which is motivated by the well-known Zariski covering families in commutative algebraic geometry.

### Definition 4.2

An ${^{H}}\mathscr {S}$-Zariski covering family is a finite family of ${^{H}}\mathscr {S}$-morphisms

4.5 $$\begin{aligned} \big \{f_{i}{:}\,X_{A[s_i^{-1}]} \longrightarrow X_{A}^{}\big \}, \end{aligned}$$

where

(i) $s_i\in A$ is an *H*-coinvariant element, i.e., $\rho ^A(s_i) =\mathbbm {1}_H^{}\otimes s_i$, for all *i*;
(ii) $f_i$ is specified by the canonical ${^{H}}\mathscr {A}_{\mathrm {fp}}$-morphism $f_i^*= \ell _{s_i}{:}\,A\rightarrow A[s_i^{-1}]$, for all *i*;
(iii) there exists a family of elements $a_i\in A$ such that $\sum _i\, a_i\,s_i =\mathbbm {1}_A^{}$.

### Example 4.3

Recall from Example 3.5 that the algebra of functions on the 2*N*-dimensional Connes–Landi sphere is given by

4.6 $$\begin{aligned} A_{\mathbb {S}^{2N}_\Theta } = F_{{\varvec{m}}_1,\ldots ,{\varvec{m}}_N,-{\varvec{m}}_1,\ldots ,-{\varvec{m}}_N,{\varvec{0}}}{\big /}I_{\mathbb {S}_{\Theta }^{2N}}. \end{aligned}$$

As the last generator $x_{2N+1}$ is *H*-coinvariant, we can define the two *H*-coinvariant elements $s_1 := \frac{1}{2}\, (\mathbbm {1}- x_{2N+1})$ and $s_{2} := \frac{1}{2}\, (\mathbbm {1}+ x_{2N+1})$. Then, $s_1 + s_2 =\mathbbm {1}$, and hence, we obtain an ${^{H}}\mathscr {S}$-Zariski covering family

4.7 $$\begin{aligned} \Big \{ f_i{:}\,X_{A_{\mathbb {S}^{2N}_\Theta }[s_i^{-1}]} \longrightarrow X_{A_{\mathbb {S}^{2N}_\Theta }}^{}\Big \}_{i=1,2} \end{aligned}$$

for the 2*N*-dimensional Connes–Landi sphere. Geometrically, $X_{A_{\mathbb {S}^{2N}_\Theta }[s_1^{-1}]}$ is the sphere with the north pole removed and similarly $X_{A_{\mathbb {S}^{2N}_\Theta }[s_2^{-1}]}$ the sphere with the south pole removed.

We now show that ${^{H}}\mathscr {S}$-Zariski covering families are stable under pullbacks.

### Proposition 4.4

The pullback of an ${^{H}}\mathscr {S}$-Zariski covering family $\{f_{i}{:}\,X_{A[s_i^{-1}]} \rightarrow X_{A}^{}\}$ along an ${^{H}}\mathscr {S}$-morphism $g{:}\,X_{B} \rightarrow X_A$ is an ${^{H}}\mathscr {S}$-Zariski covering family, i.e., the left vertical arrows of the pullback diagrams4.8define an ${^{H}}\mathscr {S}$-Zariski covering family.

### Proof

By definition, $X_{B}\times _{X_A}^{} X_{A[s_i^{-1}]} = X_{B\sqcup _A^{} A[s_i^{-1}]}$. By universality of the pushout and localization, the pushout diagram for $B\sqcup _A^{} A[s_i^{-1}]$ extends to the commutative diagram4.9It is an elementary computation to confirm that the dashed arrow in this diagram is an isomorphism by using the explicit formulas for the pushout (3.23) and localization (3.28). As a consequence, $X_{B}\times _{X_A}^{} X_{A[s_i^{-1}]} \simeq X_{B[g^*(s_i)^{-1}]}$ and the left vertical arrow in (4.8) is of the form as required in Definition 4.2 (i) and (ii). To show also item (iii) of Definition 4.2, if $a_i\in A$ is a family of elements such that $\sum _i \, a_i\,s_i=\mathbbm {1}_A^{}$, then $b_i := g^*(a_i)\in B$ is a family of elements such that $\sum _{i}\, b_i \, g^{*}(s_i) = \mathbbm {1}_B^{}$. $\square$

### Corollary 4.5

Let $\{f_{i}{:}\,X_{A[s_i^{-1}]} \rightarrow X_{A}^{}\}$ be an ${^{H}}\mathscr {S}$-Zariski covering family. Then, the pullback4.10is isomorphic to $X_{A[s_i^{-1},\, s_j^{-1}]}$, where

4.11 $$\begin{aligned} A[s_i^{-1},\, s_j^{-1}] := \big (A[s_i^{-1}]\big )[\ell _{s_i}(s_j)^{-1}] \simeq \big (A[s_j^{-1}]\big )[ \ell _{s_j}(s_i)^{-1}] \end{aligned}$$

is the localization with respect to the two *H*-coinvariant elements $s_i,s_j\in A$. The dashed arrows in (4.10) are specified by the canonical ${^{H}}\mathscr {A}_{\mathrm {fp}}$-morphisms $\ell _{s_j}{:}\,A[s_i^{-1}] \rightarrow A[s_i^{-1},\,s_j^{-1}]$ and $\ell _{s_i}{:}\,A[s_j^{-1}] \rightarrow A[s_i^{-1},\,s_j^{-1}]$.

### Proof

This follows immediately from the proof of Proposition 4.4. $\square$

### Remark 4.6

For later convenience, we shall introduce the notation4.12for the morphisms of this pullback diagram.

## Generalized toric noncommutative spaces

The category ${^{H}}\mathscr {S}$ of toric noncommutative spaces has the problem that it does not generally admit exponential objects ${X_B}^{X_A}$, i.e., objects which describe the ‘mapping space’ from $X_A$ to $X_B$. A similar problem is well known from differential geometry, where the mapping space between two finite-dimensional manifolds in general is not a finite-dimensional manifold. There is, however, a canonical procedure for extending the category ${^{H}}\mathscr {S}$ to a bigger category that admits exponential objects. We review this procedure in our case of interest.

The desired extension of ${^{H}}\mathscr {S}$ is given by the category

5.1 $$\begin{aligned} {^{H}}\mathscr {G}:= \mathrm {Sh}\big ({^{H}}\mathscr {S}\big ) \end{aligned}$$

of sheaves on ${^{H}}\mathscr {S}$ with covering families given in Definition 4.2. Recall that a sheaf on ${^{H}}\mathscr {S}$ is a functor $Y{:}\,{^{H}}\mathscr {S}^\mathrm {op}\rightarrow \mathsf {Set}$ to the category of sets (called a presheaf) that satisfies the sheaf condition: For any ${^{H}}\mathscr {S}$-Zariski covering family $\{f_i{:}\,X_{A[s_i^{-1}]} \rightarrow X_A^{}\}$, the canonical diagram5.2is an equalizer in $\mathsf {Set}$. We have used Corollary 4.5 to express the pullback of two covering morphisms by $X_{A[s_i^{-1},\,s_j^{-1}]}$. Because ${^{H}}\mathscr {S}= ({^{H}}\mathscr {A}_{\mathrm {fp}})^\mathrm {op}$ was defined as the opposite category of ${^{H}}\mathscr {A}_{\mathrm {fp}}$, it is sometimes convenient to regard a sheaf on ${^{H}}\mathscr {S}$ as a covariant functor $Y{:}\,{^{H}}\mathscr {A}_{\mathrm {fp}} \rightarrow \mathsf {Set}$. In this notation, the sheaf condition (5.2) looks like5.3We will interchangeably use these equivalent points of view. The morphisms in ${^{H}}\mathscr {G}$ are natural transformations between functors, i.e., presheaf morphisms.

We shall interpret ${^{H}}\mathscr {G}$ as a category of generalized toric noncommutative spaces. To justify this interpretation, we will show that there is a fully faithful embedding ${^{H}}\mathscr {S}\rightarrow {^{H}}\mathscr {G}$ of the category of toric noncommutative spaces into the new category. As a first step, we use the (fully faithful) Yoneda embedding ${^{H}}\mathscr {S}\rightarrow \mathrm {PSh}({^{H}}\mathscr {S})$ in order to embed ${^{H}}\mathscr {S}$ into the category of presheaves on ${^{H}}\mathscr {S}$. The Yoneda embedding is given by the functor which assigns to any object $X_A$ in ${^{H}}\mathscr {S}$ the presheaf given by the functor

5.4a $$\begin{aligned} \underline{X_A} := \mathrm {Hom}_{{^{H}}\mathscr {S}}^{}(-,X_A){:}\,{^{H}}\mathscr {S}^\mathrm {op}\longrightarrow \mathsf {Set}\end{aligned}$$

and to any ${^{H}}\mathscr {S}$-morphism $f{:}\,X_A\rightarrow X_B$ the natural transformation $\underline{f}{:}\,\underline{X_A}\rightarrow \underline{X_B}$ with components

5.4b $$\begin{aligned} \underline{f}_{X_C}^{}{:}\,\mathrm {Hom}_{{^{H}}\mathscr {S}}^{}(X_C,X_A) \longrightarrow \mathrm {Hom}_{{^{H}}\mathscr {S}}^{}(X_C,X_B),~~ g\longmapsto f\circ g, \end{aligned}$$

for all objects $X_C$ in ${^{H}}\mathscr {S}$.

### Proposition 5.1

For any object $X_{A}$ in ${^{H}}\mathscr {S}$, the presheaf $\underline{X_A}$ is a sheaf on ${^{H}}\mathscr {S}$. As a consequence, the Yoneda embedding induces a fully faithful embedding ${^{H}}\mathscr {S}\rightarrow {^{H}}\mathscr {G}$ into the category of sheaves on ${^{H}}\mathscr {S}$.

### Proof

We have to show that the functor $\underline{X_A}{:}\,{^{H}}\mathscr {S}^\mathrm {op}\rightarrow \mathsf {Set}$ satisfies the sheaf condition (5.2), or equivalently (5.3). Given any ${^{H}}\mathscr {S}$-Zariski covering family $\{f_i{:}\,X_{B[s_i^{-1}]}\rightarrow X_B^{}\}$, we therefore have to confirm that5.5is an equalizer in $\mathsf {Set}$, where we used $\underline{X_A}(X_B) = \mathrm {Hom}_{{^{H}}\mathscr {S}}^{}(X_B,X_A) = \mathrm {Hom}_{{^{H}}\mathscr {A}_{\mathrm {fp}}}^{}(A,B)$. Because the $\mathrm {Hom}$-functor $\mathrm {Hom}_{{^{H}}\mathscr {A}_{\mathrm {fp}}} (A,-){:}\,{^{H}}\mathscr {A}_{\mathrm {fp}} \rightarrow \mathsf {Set}$ preserves limits, it is sufficient to prove that5.6is an equalizer in ${^{H}}\mathscr {A}_{\mathrm {fp}}$.

Using the explicit characterization of localizations [cf. (3.28)], let us take a generic element

5.7 $$\begin{aligned} \prod _{i}\, [b_i\otimes c_i] \ \in \ \prod \limits _{i}\, B[s_i^{-1}] = \prod \limits _{i}\, B\sqcup F_{{\varvec{0}}}/(s_i\otimes x_i - \mathbbm {1}), \end{aligned}$$

where here there is no sum over the index *i* but an implicit sum of the form $[b_i\otimes c_i] = \sum _{\alpha }\, [{(b_i)}_{\alpha } \otimes {(c_{i})}_\alpha ]$ which we suppress. This is an element in the desired equalizer if and only if

5.8 $$\begin{aligned}{}[b_i\otimes c_i\otimes \mathbbm {1}] = [b_j\otimes \mathbbm {1}\otimes c_j], \end{aligned}$$

for all *i*, *j*, as equalities in $B[s_{i}^{-1},\,s_j^{-1}]$. Recalling that the relations in $B[s_{i}^{-1},\,s_j^{-1}]$ are given by $s_i\otimes x_i \otimes \mathbbm {1} =\mathbbm {1}$ and $s_j\otimes \mathbbm {1}\otimes x_j=\mathbbm {1}$, the equalities (5.8) hold if and only if $[b_i\otimes c_i] = [b\otimes \mathbbm {1}]$ with the same $b\in B$, for all *i*. Hence, (5.6) is an equalizer. $\square$

### Remark 5.2

Heuristically, Proposition 5.1 implies that the theory of toric noncommutative spaces $X_A$ together with their morphisms can be equivalently described within the category ${^{H}}\mathscr {G}$. The sheaf $\underline{X_A}$ specified by (5.4) is interpreted as the ‘functor of points’ of the toric noncommutative space $X_A$. In this interpretation, (5.4) tells us all possible ways in which any other toric noncommutative space $X_B$ may be mapped into $X_A$, which captures the geometric structure of $X_A$. A generic object *Y* in ${^{H}}\mathscr {G}$ (which we call a generalized toric noncommutative space) has a similar interpretation: The set $Y(X_B)$ tells us all possible ways in which $X_B$ is mapped into *Y*. This is formalized by Yoneda’s Lemma

5.9 $$\begin{aligned} Y(X_B) \simeq \mathrm {Hom}_{{^{H}}\mathscr {G}}(\underline{X_B},Y), \end{aligned}$$

for any object $X_B$ in ${^{H}}\mathscr {S}$ and any object *Y* in ${^{H}}\mathscr {G}$.

The advantage of the sheaf category ${^{H}}\mathscr {G}$ of generalized toric noncommutative spaces over the original category ${^{H}}\mathscr {S}$ of toric noncommutative spaces is that it has very good categorical properties, which are summarized in the notion of a Grothendieck topos, see, e.g., [22]. Most important for us are the facts that ${^{H}}\mathscr {G}$ has all (small) limits and all exponential objects. Limits in ${^{H}}\mathscr {G}$ are computed object-wise, i.e., as in presheaf categories. In particular, the product of two objects *Y*, *Z* in ${^{H}}\mathscr {G}$ is the sheaf specified by the functor $Y\times Z{:}\,{^{H}}\mathscr {S}^{\mathrm {op}} \rightarrow \mathsf {Set}$ that acts on objects as

5.10a $$\begin{aligned} (Y\times Z)(X_A) := Y(X_A)\times Z(X_A), \end{aligned}$$

where on the right-hand side $\times$ is the Cartesian product in $\mathsf {Set}$, and on morphisms $f{:}\,X_A\rightarrow X_B$ as

5.10b $$\begin{aligned} (Y\times Z)(f) := Y(f)\times Z(f){:}\,( Y\times Z)(X_B) \longrightarrow (Y\times Z) (X_A). \end{aligned}$$

The terminal object in ${^{H}}\mathscr {G}$ is the sheaf specified by the functor $\{*\}{:}\,{^{H}}\mathscr {S}^{\mathrm {op}} \rightarrow \mathsf {Set}$ that acts on objects as

5.11 $$\begin{aligned} \{*\}(X_A) := \{*\}, \end{aligned}$$

where on the right-hand side $\{*\}$ is the terminal object in $\mathsf {Set}$, i.e., a singleton set, and in the obvious way on morphisms. The fully faithful embedding ${^{H}}\mathscr {S}\rightarrow {^{H}}\mathscr {G}$ of Proposition 5.1 is limit preserving. In particular, we have

5.12 $$\begin{aligned} \underline{X_A\times X_B} = \underline{X_A}\times \underline{X_B},\quad \underline{X_{\mathbb {K}}} = \{*\}, \end{aligned}$$

for all objects $X_A,X_B$ in ${^{H}}\mathscr {S}$ and the terminal object $X_{\mathbb {K}}$ in ${^{H}}\mathscr {S}$. Here $\mathbb {K}$ is the ${^{H}}\mathscr {A}_{\mathrm {fp}}$-algebra with trivial left *H*-coaction $c\mapsto \mathbbm {1}_H\otimes c$, i.e., the initial object in ${^{H}}\mathscr {A}_{\mathrm {fp}}$.

The exponential objects in ${^{H}}\mathscr {G}$ are constructed as follows: Given two objects *Y*, *Z* in ${^{H}}\mathscr {G}$, the exponential object $Z^Y$ is the sheaf specified by the functor $Z^Y{:}\,{^{H}}\mathscr {S}^{\mathrm {op}} \rightarrow \mathsf {Set}$ that acts on objects as

5.13a $$\begin{aligned} Z^Y(X_A) := \mathrm {Hom}_{{^{H}}\mathscr {G}}\big (\underline{X_A} \times Y, Z\big ), \end{aligned}$$

and on morphisms $f{:}\,X_A\rightarrow X_B$ as

5.13b $$\begin{aligned} Z^Y(f){:}\,Z^Y(X_B)\longrightarrow Z^Y(X_A),~~g\longmapsto g\circ ( \underline{f}\times \mathrm {id}_{Y}), \end{aligned}$$

where $\underline{f}{:}\,\underline{X_A}\rightarrow \underline{X_B}$ is the ${^{H}}\mathscr {G}$-morphism specified by (5.4). The formation of exponential objects is functorial, i.e., there are obvious functors $(-)^Y{:}\,{^{H}}\mathscr {G}\rightarrow {^{H}}\mathscr {G}$ and $Z^{(-)}{:}\,{^{H}}\mathscr {G}^\mathrm {op}\rightarrow {^{H}}\mathscr {G}$, for all objects *Y*, *Z* in ${^{H}}\mathscr {G}$. Moreover, there are natural isomorphisms

5.14 $$\begin{aligned} \{*\}^Y\simeq \{*\},~~~Z^{\{*\}}\simeq Z,~~~(Z\times Z^\prime )^Y\simeq Z^Y\times {Z^\prime }\,^Y,~~~ Z^{Y\times Y^\prime } \simeq {(Z^Y)}^{Y^\prime }, \end{aligned}$$

for all objects $Y,Y^\prime ,Z,Z^\prime$ in ${^{H}}\mathscr {G}$.

Given two ordinary toric noncommutative spaces $X_A$ and $X_B$, i.e., objects in ${^{H}}\mathscr {S}$, we can form the exponential object $\underline{X_B}^{\underline{X_A}}$ in the category of *generalized* toric noncommutative spaces ${^{H}}\mathscr {G}$. The interpretation of $\underline{X_B}^{\underline{X_A}}$ is as the ‘space of maps’ from $X_A$ to $X_B$. In the present situation, the explicit description (5.13) of exponential objects may be simplified via

5.15 $$\begin{aligned} \underline{X_B}^{\underline{X_A}}(X_C)&= \mathrm {Hom}_{{^{H}}\mathscr {G}}\big ( \underline{X_C}\times \underline{X_A},\,\underline{X_B}\, \big )\nonumber \\&= \mathrm {Hom}_{{^{H}}\mathscr {G}}\big ( \underline{X_C\times X_A},\,\underline{X_B}\, \big )\nonumber \\&\simeq \mathrm {Hom}_{{^{H}}\mathscr {S}}\big (X_C\times X_A,X_B\big ) \nonumber \\&= \mathrm {Hom}_{{^{H}}\mathscr {A}_{\mathrm {fp}}}(B, C\sqcup A). \end{aligned}$$

In the first step, we have used (5.13), in the second step (5.12), and the third step is due to Yoneda’s Lemma. Hence,

5.16 $$\begin{aligned} \underline{X_B}^{\underline{X_A}} \simeq \mathrm {Hom}_{{^{H}}\mathscr {A}_{\mathrm {fp}}}(B, - \sqcup A){:}\,{^{H}}\mathscr {A}_{\mathrm {fp}}\longrightarrow \mathsf {Set}\end{aligned}$$

can be expressed in terms of ${^{H}}\mathscr {A}_{\mathrm {fp}}$-morphisms.

### Example 5.3

To illustrate the differences between the exponential objects $\underline{X_B}^{\underline{X_A}}$ in ${^{H}}\mathscr {G}$ and the $\mathrm {Hom}$-sets $\mathrm {Hom}_{{^{H}}\mathscr {S}}(X_A,X_B)$, let us consider the simplest example where $A=B=F_{{\varvec{m}}}$, for some ${\varvec{m}}\ne {\varvec{0}}$ in $\mathbb {Z}^n$. In this case, the $\mathrm {Hom}$-set is given by

5.17 $$\begin{aligned} \mathrm {Hom}_{{^{H}}\mathscr {S}}(X_{F_{{\varvec{m}}}},X_{F_{{\varvec{m}}}}) = \mathrm {Hom}_{{^{H}}\mathscr {A}_{\mathrm {fp}}} (F_{{\varvec{m}}},F_{{\varvec{m}}}) \simeq \mathbb {K}, \end{aligned}$$

because by *H*-equivariance any ${^{H}}\mathscr {A}_{\mathrm {fp}}$-morphism $\kappa {:}\,F_{{\varvec{m}}}\rightarrow F_{{\varvec{m}}}$ is of the form $\kappa (x) = c\,x$, for some $c\in \mathbb {K}$; here *x* denotes the generator of $F_{{\varvec{m}}}$, whose left *H*-coaction is by definition $\rho ^{F_{{\varvec{m}}}}(x) = t_{{\varvec{m}}}\otimes x$. On the other hand, the exponential object $\underline{X_{F_{{\varvec{m}}}}}^{\underline{X_{F_{{\varvec{m}}}}}}$ is a functor from ${^{H}}\mathscr {S}^{\mathrm {op}} = {^{H}}\mathscr {A}_{\mathrm {fp}}$ to $\mathsf {Set}$, and hence, it gives us a set for any test ${^{H}}\mathscr {A}_{\mathrm {fp}}$-algebra *A*. Using (5.16), we obtain

5.18 $$\begin{aligned} \underline{X_{F_{{\varvec{m}}}}}^{\underline{X_{F_{{\varvec{m}}}}}}(A) = \mathrm {Hom}_{{^{H}}\mathscr {A}_{\mathrm {fp}}}(F_{{\varvec{m}}}, A \sqcup F_{{\varvec{m}}}). \end{aligned}$$

For the initial object $A = \mathbb {K}$ in ${^{H}}\mathscr {A}_{\mathrm {fp}}$, we recover the $\mathrm {Hom}$-set

5.19 $$\begin{aligned} \underline{X_{F_{{\varvec{m}}}}}^{\underline{X_{F_{{\varvec{m}}}}}}(\mathbb {K}) = \mathrm {Hom}_{{^{H}}\mathscr {S}}(X_{F_{{\varvec{m}}}},X_{F_{{\varvec{m}}}})\simeq \mathbb {K}. \end{aligned}$$

Let us now take $A_{\mathbb {T}_{\Theta }} = F_{-{\varvec{m}},{\varvec{m}}}/(y^*\, y -\mathbbm {1})$ to be the toric noncommutative circle, see Example 3.5. We write $y^{-1}:=y^*$ in $A_{\mathbb {T}_{\Theta }}$ and recall that the left *H*-coaction is given by $\rho ^{F_{-{\varvec{m}},{\varvec{m}}} }(y) = t_{-{\varvec{m}}}\otimes y$ and $\rho ^{F_{-{\varvec{m}},{\varvec{m}}} }(y^{-1}) = t_{{\varvec{m}}}\otimes y^{-1}$. We then obtain an isomorphism of sets

5.20 $$\begin{aligned} \underline{X_{F_{{\varvec{m}}}}}^{\underline{X_{F_{{\varvec{m}}}}}}(A_{\mathbb {T}_{\Theta }}) = \mathrm {Hom}_{{^{H}}\mathscr {A}_{\mathrm {fp}}}\left( F_{{\varvec{m}}}, A_{\mathbb {T}_{\Theta }}\sqcup F_{{\varvec{m}}}\right) \simeq F_{{\varvec{m}}}, \end{aligned}$$

because elements $a\in F_{{\varvec{m}}}$, i.e., polynomials $a= \sum _{j}\, c_j\,x^j$, for $c_j\in \mathbb {K}$, are in bijection with ${^{H}}\mathscr {A}_{\mathrm {fp}}$-morphisms $\kappa {:}\,F_{{\varvec{m}}}\rightarrow A_{\mathbb {T}_{\Theta }} \sqcup F_{{\varvec{m}}}$ via

5.21 $$\begin{aligned} \kappa (x) = \sum _{j}\, c_j ~ y^{j-1}\otimes x^j. \end{aligned}$$

By construction, each summand on the right-hand side has left *H*-coaction $\rho ^{F_{-{\varvec{m}},{\varvec{m}}} }(y^{j-1}\otimes x^j) = t_{-(j-1)\,{\varvec{m}}}\,t_{j\,{\varvec{m}}} \otimes y^{j-1}\otimes x^j = t_{{\varvec{m}}}\otimes y^{j-1}\otimes x^j$. Heuristically, this means that the exponential object $\underline{X_{F_{{\varvec{m}}}}}^{\underline{X_{F_{{\varvec{m}}}}}}$ captures all polynomial maps $F_{{\varvec{m}}}\rightarrow F_{{\varvec{m}}}$, while the $\mathrm {Hom}$-set $\mathrm {Hom}_{{^{H}}\mathscr {S}}(X_{F_{{\varvec{m}}}},X_{F_{{\varvec{m}}}})$ captures only those that are *H*-equivariant which in the present case are the linear maps $x\mapsto c\,x$. Similar results hold for generic exponential objects $\underline{X_B}^{\underline{X_A}}$ in ${^{H}}\mathscr {G}$; in particular, their global points $\underline{X_B}^{\underline{X_A}}(X_\mathbb {K})$ coincide with the $\mathrm {Hom}$-sets $\mathrm {Hom}_{{^{H}}\mathscr {S}}(X_A,X_B)$ while their generalized points $\underline{X_B}^{\underline{X_A}}(X_C)$, for $X_C$ an object in ${^{H}}\mathscr {S}$, capture additional maps.

## Automorphism groups

Associated with any object, $X_A$ in ${^{H}}\mathscr {S}$ is its exponential object $\underline{X_A}^{\underline{X_A}}$ in ${^{H}}\mathscr {G}$ which describes the ‘space of maps’ from $X_A$ to itself. The object $\underline{X_A}^{\underline{X_A}}$ has a distinguished point (called the identity) given by the ${^{H}}\mathscr {G}$-morphism

6.1a $$\begin{aligned} e{:}\,\{*\} \longrightarrow \underline{X_A}^{\underline{X_A}} \end{aligned}$$

that is specified by the natural transformation with components

6.1b $$\begin{aligned} e_{X_B}{:}\,\{*\} \longrightarrow \mathrm {Hom}_{{^{H}}\mathscr {S}}\big (X_B\times X_A,X_A\big ),~~*\longmapsto \big (\pi _{2}{:}\,X_B\times X_A \rightarrow X_A\big )~ \end{aligned}$$

given by the canonical projection ${^{H}}\mathscr {S}$-morphisms of the product. Under the Yoneda bijections $\mathrm {Hom}_{{^{H}}\mathscr {G}}(\{*\}, \underline{X_A}^{\underline{X_A}}) \simeq \underline{X_A}^{\underline{X_A}}(\{*\})\simeq \mathrm {Hom}_{{^{H}}\mathscr {S}}(X_A,X_A)$, *e* is mapped to the identity ${^{H}}\mathscr {S}$-morphism $\mathrm {id}_{X_A}$. Moreover, there is a composition ${^{H}}\mathscr {G}$-morphism

6.2a $$\begin{aligned} \bullet {:}\,\underline{X_A}^{\underline{X_A}} \times \underline{X_A}^{\underline{X_A}}\longrightarrow \underline{X_A}^{\underline{X_A}} \end{aligned}$$

that is specified by the natural transformation with components

6.2b $$\begin{aligned} \bullet _{X_B}:\mathrm {Hom}_{{^{H}}\mathscr {S}}\big (X_B\times X_A,X_A\big )\times \mathrm {Hom}_{{^{H}}\mathscr {S}}\big (X_B\times X_A,X_A\big )&\longrightarrow \mathrm {Hom}_{{^{H}}\mathscr {S}}\big (X_B\times X_A,X_A\big ),\nonumber \\ (g,h)&\longmapsto g\bullet _{X_B} h \end{aligned}$$

defined by

6.2c $$\begin{aligned} g\bullet _{X_B} h := g\circ (\mathrm {id}_{X_B}\times h)\circ (\mathrm {diag}_{X_B}\times \mathrm {id}_{X_A}){:}\,X_B\times X_A\longrightarrow X_A, \end{aligned}$$

for all ${^{H}}\mathscr {S}$-morphisms $g,h{:}\,X_B\times X_A\rightarrow X_A$. The diagonal ${^{H}}\mathscr {S}$-morphism $\mathrm {diag}_{X_B}{:}\,X_B\rightarrow X_B\times X_B$ is defined as usual via universality of products by6.3The object $\underline{X_A}^{\underline{X_A}}$ together with the identity (6.1) and composition ${^{H}}\mathscr {G}$-morphisms (6.2) is a monoid object in ${^{H}}\mathscr {G}$: It is straightforward to verify that the ${^{H}}\mathscr {G}$-diagrams 6.4aand6.4b commute. Notice that $\underline{X_A}^{\underline{X_A}}$ is not a group object in ${^{H}}\mathscr {G}$ because, loosely speaking, generic maps do not have an inverse. We may, however, construct the ‘subobject of invertible maps’ (in a suitable sense to be detailed below) of the monoid object $\underline{X_A}^{\underline{X_A}}\,$, which then becomes a group object in ${^{H}}\mathscr {G}$ called the automorphism group $\mathrm {Aut}(X_A)$ of $X_A$.

Let us apply the fully faithful functor $\mathrm {Sh}({^{H}}\mathscr {S}) \rightarrow \mathrm {PSh}({^{H}}\mathscr {S})$ (which assigns to sheaves their underlying presheaves) on the monoid object $( \underline{X_A}^{\underline{X_A}},\bullet , e)$ in ${^{H}}\mathscr {G}= \mathrm {Sh}({^{H}}\mathscr {S})$ to obtain the monoid object $( \underline{X_A}^{\underline{X_A}},\bullet , e)$ in $\mathrm {PSh}({^{H}}\mathscr {S})$, denoted with abuse of notation by the same symbol. This monoid object in $\mathrm {PSh}({^{H}}\mathscr {S})$ may be equivalently regarded as a functor ${^{H}}\mathscr {S}^{\mathrm {op}} \rightarrow \mathsf {Monoid}$ with values in the category of ordinary $\mathsf {Set}$-valued monoids (i.e., monoid objects in the category $\mathsf {Set}$). The functor assigns to any object $X_B$ in ${^{H}}\mathscr {S}$ the monoid

6.5a $$\begin{aligned} \Big (\underline{X_A}^{\underline{X_A}}(X_B), \bullet _{X_B}, e_{X_B}\Big ) \end{aligned}$$

and to any ${^{H}}\mathscr {S}$-morphism $f{:}\,X_B\rightarrow X_C$ the monoid morphism

6.5b $$\begin{aligned} \underline{X_A}^{\underline{X_A}}(f){:}\, \Big (\underline{X_A}^{\underline{X_A}}(X_C), \bullet _{X_C}, e_{X_C}\Big ) \longrightarrow \Big (\underline{X_A}^{\underline{X_A}}(X_B), \bullet _{X_B}, e_{X_B}\Big ). \end{aligned}$$

For any object $X_B$ in ${^{H}}\mathscr {S}$, we define $\mathrm {Aut}(X_A)(X_B)$ to be the subset of elements $g\in \underline{X_A}^{\underline{X_A}}(X_B)$ for which there exists $g^{-1}\in \underline{X_A}^{\underline{X_A}}(X_B)$ such that

6.6 $$\begin{aligned} g\bullet _{X_B} g^{-1} = g^{-1}\bullet _{X_B} g = e_{X_B}. \end{aligned}$$

Because the inverse of an element in a monoid (if it exists) is always unique, it follows that any element $g\in \mathrm {Aut}(X_A)(X_B)$ has a unique inverse $g^{-1}\in \mathrm {Aut}(X_A)(X_B)$, and that the inverse of $g^{-1}$ is *g*. The monoid structure on $\underline{X_A}^{\underline{X_A}}(X_B)$ induces to $\mathrm {Aut}(X_A)(X_B)$, because the inverse of $e_{X_B}$ is $e_{X_B}$ itself and the inverse of $g\bullet _{X_B} h$ is $(g\bullet _{X_B} h)^{-1} = h^{-1}\bullet _{X_B} g^{-1}$. Denoting by

6.7 $$\begin{aligned} \mathrm {inv}_{X_B}{:}\,\mathrm {Aut}(X_A)(X_B)\longrightarrow \mathrm {Aut}(X_A)(X_B),~~g\longmapsto g^{-1} \end{aligned}$$

the map that assigns the inverse, we obtain for any object $X_B$ in ${^{H}}\mathscr {S}$ a group

6.8a $$\begin{aligned} \big (\mathrm {Aut}(X_A)(X_B), \bullet _{X_B}, e_{X_B},\mathrm {inv}_{X_B}\big ). \end{aligned}$$

The monoid morphism $\underline{X_A}^{\underline{X_A}}(f)$ in (6.5) induces a group morphism which we denote by

6.8b $$\begin{aligned}&\mathrm {Aut}(X_A)(f){:}\,\big (\mathrm {Aut}(X_A)(X_C), \bullet _{X_C}, e_{X_C},\mathrm {inv}_{X_C}\big )\nonumber \\&\quad \longrightarrow \big (\mathrm {Aut}(X_A)(X_B), \bullet _{X_B}, e_{X_B},\mathrm {inv}_{X_B}\big ). \end{aligned}$$

Hence, we have constructed a functor ${^{H}}\mathscr {S}^{\mathrm {op}} \rightarrow \mathsf {Group}$ with values in the category of ordinary $\mathsf {Set}$-valued groups (i.e., group objects in $\mathsf {Set}$), which we can equivalently regard as a group object $(\mathrm {Aut}(X_A),\bullet ,e,\mathrm {inv})$ in the category $\mathrm {PSh}({^{H}}\mathscr {S})$. Notice further that $\mathrm {Aut}(X_A)$ is a subobject of $\underline{X_A}^{\underline{X_A}}$ in the category $\mathrm {PSh}({^{H}}\mathscr {S})$.

### Proposition 6.1

For any object $X_A$ in ${^{H}}\mathscr {S}$, the presheaf $\mathrm {Aut}(X_A)$ satisfies the sheaf condition (5.2). In particular, $(\mathrm {Aut}(X_A),\bullet ,e,\mathrm {inv})$ is the subobject of invertibles of the monoid object $(\underline{X_A}^{\underline{X_A}},\bullet ,e)$ in ${^{H}}\mathscr {G}$ and hence a group object in ${^{H}}\mathscr {G}$.

### Proof

Given any ${^{H}}\mathscr {S}$-Zariski covering family $\{f_i{:}\,X_{B[s_i^{-1}]} \rightarrow X_{B}\}$, we have to show that6.9is an equalizer in $\mathsf {Set}$. Recalling that $\mathrm {Aut}(X_A)$ is the subpresheaf of the sheaf $\underline{X_A}^{\underline{X_A}}$ specified by the invertibility conditions (6.6), an element in $\prod _{i}\, \mathrm {Aut}(X_A)(X_{B[s_i^{-1}]})$ can be represented by an element

6.10 $$\begin{aligned} \prod _{i} \, g_i \ \in \ \prod \limits _{i}\, \underline{X_A}^{\underline{X_A}} \big (X_{B[s_i^{-1}]} \big ), \end{aligned}$$

such that each $g_i$ has an inverse $g_i^{-1}\in \underline{X_A}^{\underline{X_A}}\big (X_{B[s_i^{-1}]} \big )$ in the sense that

6.11 $$\begin{aligned} g_i\bullet _{X_{B[s_i^{-1}]}} g_i^{-1} = g_i^{-1}\bullet _{X_{B[s_i^{-1}]}} g_i= e_{X_{B[s_i^{-1}]}}. \end{aligned}$$

This element is in the desired equalizer if and only if

6.12 $$\begin{aligned} \underline{X_A}^{\underline{X_A}}(f_{i;j})\big ( g_j\big ) = \underline{X_A}^{\underline{X_A}}(f_{j;i})\big ( g_i\big ), \end{aligned}$$

for all *i*, *j*, where we used the compact notation $f_{i;j}$ introduced in (4.12). Because $\underline{X_A}^{\underline{X_A}}$ is a sheaf, we can represent $\prod _{i} \, g_i$ by the element $g\in \underline{X_A}^{\underline{X_A}}(X_B)$ that is uniquely specified by $\underline{X_A}^{\underline{X_A}}(f_i)(g) = g_i$, for all *i*.

We have to show that $g\in \mathrm {Aut}(X_A)(X_B)\subseteq \underline{X_A}^{\underline{X_A}}(X_B)$, i.e., that there exists $g^{-1}\in \underline{X_A}^{\underline{X_A}}(X_B)$ such that $g\bullet _{X_B}g^{-1}= g^{-1}\bullet _{X_B} g = e_{X_B}$. Since $\underline{X_A}^{\underline{X_A}}(f_{i;j})$ and $\underline{X_A}^{\underline{X_A}}(f_{j;i})$ are monoid morphisms, both sides of the equality (6.12) are invertible and the inverse is given by

6.13 $$\begin{aligned} \underline{X_A}^{\underline{X_A}}(f_{i;j})\big ( g_j^{-1}\big ) = \underline{X_A}^{\underline{X_A}}(f_{j;i})\big ( g_i^{-1}\big ). \end{aligned}$$

Using again the property that $\underline{X_A}^{\underline{X_A}}$ is a sheaf, we can represent $\prod _{i} \, g^{-1}_i$ by the element $\tilde{g} \in \underline{X_A}^{\underline{X_A}}(X_B)$ that is uniquely specified by $\underline{X_A}^{\underline{X_A}}(f_i)(\tilde{g}) = g^{-1}_i$, for all *i*. It is now easy to check that $\tilde{g}$ is the inverse of *g*: Using once more the property that $\underline{X_A}^{\underline{X_A}}(f_i)$ are monoid morphisms, we obtain $\underline{X_A}^{\underline{X_A}}(f_i)(\tilde{g}\bullet _{X_B} g)= g_i^{-1}\bullet _{X_{B[s_i^{-1}]}} g_i = e_{X_{B[s_i^{-1}]}}$ and similarly $\underline{X_A}^{\underline{X_A}}(f_i)(g\bullet _{X_B} \tilde{g})= e_{X_{B[s_i^{-1}]}}$, for all *i*. Because $\underline{X_A}^{\underline{X_A}}$ is a sheaf, this implies $\tilde{g}\bullet _{X_B} g = g\bullet _{X_B} \tilde{g} = e_{X_B}$ and hence that $\tilde{g} = g^{-1}$. $\square$

## Lie algebras of automorphism groups

The category ${^{H}}\mathscr {G}$ of generalized toric noncommutative spaces has a distinguished object $\underline{K}:=\underline{X_{F_{{\varvec{0}}}}}$, where $F_{{\varvec{0}}}$ is the free ${^{H}}\mathscr {A}_{\mathrm {fp}}$-algebra with one coinvariant generator *x*, i.e., $x\mapsto \mathbbm {1}_H\otimes x$. We call $\underline{K}$ the line object as it describes the toric noncommutative line. The line object $\underline{K}$ is a ring object in ${^{H}}\mathscr {G}$: The sum ${^{H}}\mathscr {G}$-morphism $+{:}\,\underline{K}\times \underline{K}\rightarrow \underline{K}$ is induced (via going opposite and the Yoneda embedding) by the ${^{H}}\mathscr {A}_{\mathrm {fp}}$-morphism $F_{{\varvec{0}}} \rightarrow F_{{\varvec{0}}}\sqcup F_{{\varvec{0}}},~x\mapsto x\otimes \mathbbm {1} + \mathbbm {1}\otimes x$. The multiplication ${^{H}}\mathscr {G}$-morphism $\cdot {:}\,\underline{K}\times \underline{K}\rightarrow \underline{K}$ is induced by the ${^{H}}\mathscr {A}_{\mathrm {fp}}$-morphism $F_{{\varvec{0}}} \rightarrow F_{{\varvec{0}}}\sqcup F_{{\varvec{0}}},~x\mapsto x\otimes x$. The (additive) zero element is the ${^{H}}\mathscr {G}$-morphism $0{:}\,\{*\}\rightarrow \underline{K}$ induced by the ${^{H}}\mathscr {A}_{\mathrm {fp}}$-morphism $F_{{\varvec{0}}}\rightarrow \mathbb {K},~x\mapsto 0\in \mathbb {K}$, and the (multiplicative) unit element is the ${^{H}}\mathscr {G}$-morphism $1{:}\,\{*\}\rightarrow \underline{K}$ induced by the ${^{H}}\mathscr {A}_{\mathrm {fp}}$-morphism $F_{{\varvec{0}}}\rightarrow \mathbb {K},~x\mapsto 1\in \mathbb {K}$. Finally, the additive inverse ${^{H}}\mathscr {G}$-morphism $\mathrm {inv}_{+}{:}\,\underline{K}\rightarrow \underline{K}$ is induced by the ${^{H}}\mathscr {A}_{\mathrm {fp}}$-morphism $F_{{\varvec{0}}}\rightarrow F_{{\varvec{0}}},~x\mapsto -x$. It is straightforward, but slightly tedious, to confirm that these structures make $\underline{K}$ into a ring object in ${^{H}}\mathscr {G}$.

### Remark 7.1

Regarding the line object as a functor $\underline{K}{:}\,{^{H}}\mathscr {S}^\mathrm {op}\rightarrow \mathsf {Set}$, it assigns to an object $X_B$ in ${^{H}}\mathscr {S}$ the set

7.1a $$\begin{aligned} \underline{K}(X_B)= \mathrm {Hom}_{{^{H}}\mathscr {S}}(X_B, K) = \mathrm {Hom}_{{^{H}}\mathscr {A}_{\mathrm {fp}}}(F_{{\varvec{0}}}, B) \simeq B^{{\varvec{0}}}, \end{aligned}$$

where $B^{{\varvec{0}}} := \{b\in B{:}\,\rho ^B(b)=\mathbbm {1}_H\otimes b \}$ is the set of coinvariants; in the last step, we have used the fact that $F_{{\varvec{0}}}$ is the free ${^{H}}\mathscr {A}_{\mathrm {fp}}$-algebra with one coinvariant generator; hence, ${^{H}}\mathscr {A}_{\mathrm {fp}}$-morphisms $F_{{\varvec{0}}} \rightarrow B$ are in bijection with $B^{{\varvec{0}}}$. To an ${^{H}}\mathscr {S}$-morphism $f{:}\,X_B\rightarrow X_{B^\prime }$, it assigns the restriction of $f^*{:}\,B^\prime \rightarrow B$ to coinvariants, i.e.,

7.1b $$\begin{aligned} \underline{K}(f) = f^*{:}\,{B^\prime }\,^{{\varvec{0}}} \longrightarrow B^{{\varvec{0}}}. \end{aligned}$$

The ${^{H}}\mathscr {A}_{\mathrm {fp}}$-algebra structure on $B,B^\prime$ induces a (commutative) ring structure on $B^{{\varvec{0}}},{B^\prime }\,^{{\varvec{0}}}$ and $f^*$ preserves this ring structure. Hence, we have obtained a functor ${^{H}}\mathscr {S}^{\mathrm {op}}\rightarrow \mathsf {CRing}$ with values in the category of commutative rings (in $\mathsf {Set}$), which is an equivalent way to describe the ring object structure on $\underline{K}$ introduced above.

The ${^{H}}\mathscr {A}_{\mathrm {fp}}$-morphism $F_{{\varvec{0}}} \rightarrow F_{{\varvec{0}}}/(x^2)$ given by the quotient map induces a monomorphism $\underline{D} := \underline{X_{F_{{\varvec{0}}}/(x^2)}} \rightarrow \underline{X_{F_{{\varvec{0}}}}}= \underline{K}$ in ${^{H}}\mathscr {G}$. The zero element, sum and additive inverse of $\underline{K}$ induce to $\underline{D}$, i.e., we obtain ${^{H}}\mathscr {G}$-morphisms $0{:}\,\{*\}\rightarrow \underline{D}$, $+{:}\,\underline{D}\times \underline{D}\rightarrow \underline{D}$ and $\mathrm {inv}_{+}{:}\,\underline{D}\rightarrow \underline{D}$ which give $\underline{D}$ the structure of an Abelian group object in ${^{H}}\mathscr {G}$. Moreover, $\underline{D}$ is a $\underline{K}$-module object in ${^{H}}\mathscr {G}$ with left $\underline{K}$-action ${^{H}}\mathscr {G}$-morphism $\cdot {:}\,\underline{K}\times \underline{D}\rightarrow \underline{D}$ induced by the ${^{H}}\mathscr {A}_{\mathrm {fp}}$-morphism $F_{{\varvec{0}}}/(x^2) \rightarrow F_{{\varvec{0}}}\sqcup F_{{\varvec{0}}}/(x^2),~ x\mapsto x\otimes x$. Heuristically, $\underline{D}$ describes the infinitesimal neighborhood of 0 in $\underline{K}$, i.e., $\underline{D}$ is an infinitesimally short line, so short that functions on $\underline{D}$ (which are described by $F_{{\varvec{0}}}/(x^2)$) are polynomials of degree 1.

Following the ideas of synthetic (differential) geometry [19, 21, 26], we may use $\underline{D}$ to define the tangent bundle of a generalized toric noncommutative space.

### Definition 7.2

Let *Y* be any object in ${^{H}}\mathscr {G}$. The (total space of) tangent bundle of *Y* is the exponential object

7.2a $$\begin{aligned} TY := Y^{\underline{D}} \end{aligned}$$

in ${^{H}}\mathscr {G}$. The projection ${^{H}}\mathscr {G}$-morphism is

7.2b $$\begin{aligned} \pi := Y^{0{:}\,\{*\} \rightarrow \underline{D}}{:}\,TY = Y^{\underline{D}} \longrightarrow Y^{\{*\}} \simeq Y, \end{aligned}$$

where we use the property that $Y^{(-)}{:}\,{^{H}}\mathscr {G}^\mathrm {op}\rightarrow {^{H}}\mathscr {G}$ is a functor.

### Remark 7.3

Focusing on the underlying functors $Y{:}\,{^{H}}\mathscr {S}^\mathrm {op}\rightarrow \mathsf {Set}$ of objects *Y* in ${^{H}}\mathscr {G}$, there is an equivalent, and practically useful, characterization of the tangent bundle *TY*. By Yoneda’s Lemma and the fact that the exponential $(-)^{\underline{D}}{:}\,{^{H}}\mathscr {G}\rightarrow {^{H}}\mathscr {G}$ is the right adjoint of the product $-\times \underline{D}{:}\,{^{H}}\mathscr {G}\rightarrow {^{H}}\mathscr {G}$, there are natural isomorphisms

7.3 $$\begin{aligned} TY = Y^{\underline{D}} \simeq \mathrm {Hom}_{{^{H}}\mathscr {G}}\big (\underline{(-)}, Y^{\underline{D}}\big )\simeq \mathrm {Hom}_{{^{H}}\mathscr {G}}\big ( \underline{(-)}\times \underline{D}, Y\big ) \simeq Y\big (-\times D\big ). \end{aligned}$$

Hence, the set $TY(X_B)$ at stage $X_B$ is simply given by $Y(X_B\times D)$ at stage $X_B\times D$. The components of the projection then read as

7.4 $$\begin{aligned} \pi _{X_B} = Y(\mathrm {id}_{X_B}\times 0){:}\,Y(X_B\times D) \longrightarrow Y(X_B), \end{aligned}$$

where $\mathrm {id}_{X_B}\times 0{:}\,X_B\simeq X_B\times \{*\} \rightarrow X_B\times D$ is the product of the ${^{H}}\mathscr {S}$-morphisms $\mathrm {id}_{X_B}{:}\,X_B\rightarrow X_B$ and $0:\{*\} \rightarrow D$.

We shall now study in more detail the tangent bundle

7.5 $$\begin{aligned} \pi {:}\,T\mathrm {Aut}(X_A) \longrightarrow \mathrm {Aut}(X_A)~ \end{aligned}$$

of the automorphism group of some object $X_A$ in ${^{H}}\mathscr {S}$. We are particularly interested in the fiber $T_e\mathrm {Aut}(X_A)$ of this bundle over the identity $e{:}\,\{*\} \rightarrow \mathrm {Aut}(X_A)$, because it defines the Lie algebra of $\mathrm {Aut}(X_A)$. The fiber $T_e\mathrm {Aut}(X_A)$ is defined as the pullback7.6in ${^{H}}\mathscr {G}$. In particular, $T_e\mathrm {Aut}(X_A)$ is an object in ${^{H}}\mathscr {G}$.

Using the perspective explained in Remark 7.3, we obtain

7.7 $$\begin{aligned} T\mathrm {Aut}(X_A)(X_B) = \mathrm {Aut}(X_A)\big (X_B\times D\big ), \end{aligned}$$

for all objects $X_B$ in ${^{H}}\mathscr {S}$. The pullback (7.6) then introduces a further condition

7.8 $$\begin{aligned} T_e\mathrm {Aut}(X_A)(X_B) = \left\{ g\in \mathrm {Aut}(X_A)\big (X_B\times D\big ){:}\,\mathrm {Aut}(X_A)(\mathrm {id}_{X_B}\times 0) (g) = e_{X_{B}}\right\} , \end{aligned}$$

for all objects $X_B$ in ${^{H}}\mathscr {S}$. Using (6.6) and (5.16), it follows that any $g\in \mathrm {Aut}(X_A)(X_B\times D)$ is an ${^{H}}\mathscr {A}_{\mathrm {fp}}$-morphism $A\rightarrow B\sqcup F_{{\varvec{0}}}/(x^2) \sqcup A$ satisfying the invertibility condition imposed in (6.6). For our purposes, it is more convenient to equivalently regard *g* as an ${^{H}}\mathscr {A}_{\mathrm {fp}}$-morphism $g{:}\,A\rightarrow F_{{\varvec{0}}}/(x^2)\sqcup B \sqcup A$ with target $F_{{\varvec{0}}}/(x^2)\sqcup B \sqcup A$ instead of $B\sqcup F_{{\varvec{0}}}/(x^2) \sqcup A$ (flipping $F_{{\varvec{0}}}/(x^2)$ and *B* is the usual flip map because the left *H*-coaction on $F_{{\varvec{0}}}/(x^2)$ is trivial). Because any element in $F_{{\varvec{0}}}/(x^2)$ is of the form $c_0 + c_1\,x$, for some $c_0,c_1\in \mathbb {K}$, we obtain two ${^{H}}\mathscr {M}$-morphisms $g_0,g_1{:}\,A\rightarrow B\sqcup A$ which are characterized uniquely by

7.9 $$\begin{aligned} g(a) =\mathbbm {1}_{F_{{\varvec{0}}}/(x^2)}\otimes g_0(a) + x\otimes g_1(a), \end{aligned}$$

for all $a\in A$. Since $g{:}\,A\rightarrow F_{{\varvec{0}}}/(x^2)\sqcup B \sqcup A$ is an ${^{H}}\mathscr {A}_{\mathrm {fp}}$-morphism, it follows that $g_0{:}\,A\rightarrow B\sqcup A$ is an ${^{H}}\mathscr {A}_{\mathrm {fp}}$-morphism and that $g_1{:}\,A\rightarrow B\sqcup A$ satisfies the condition

7.10 $$\begin{aligned} g_1(a\,a^\prime ) = g_1(a)\,g_0(a^\prime ) + g_0(a)\,g_1(a^\prime ), \end{aligned}$$

for all $a,a^\prime \in A$. From (7.8), it follows that $g\in T_e\mathrm {Aut}(X_A)(X_B)$ if and only if $g_0 = \iota _2{:}\,A\rightarrow B\sqcup A,~a\mapsto \mathbbm {1}_B\otimes a$ is the canonical inclusion for the coproduct. Then, (7.10) simplifies to

7.11 $$\begin{aligned} g_1(a\,a^\prime ) = g_1(a)\,(\mathbbm {1}_B\otimes a^\prime )+ (\mathbbm {1}_B\otimes a)\, g_1(a^\prime ), \end{aligned}$$

for all $a,a^\prime \in A$. Notice that (7.11) is the Leibniz rule for the *A*-bimodule structure on $B\sqcup A$ that is induced by the ${^{H}}\mathscr {A}_{\mathrm {fp}}$-algebra structure on $B\sqcup A$ and the inclusion ${^{H}}\mathscr {A}_{\mathrm {fp}}$-morphism $\iota _2{:}\,A\rightarrow B\sqcup A$.

### Lemma 7.4

Let $g{:}\,A\rightarrow F_{{\varvec{0}}}/(x^2) \sqcup B \sqcup A$ be any ${^{H}}\mathscr {A}_{\mathrm {fp}}$-morphism such that $g_0 = \iota _2{:}\,A\rightarrow B\sqcup A$ in the notation of (7.9). Then, the ${^{H}}\mathscr {A}_{\mathrm {fp}}$-morphism $\tilde{g}{:}\, A\rightarrow F_{{\varvec{0}}}/(x^2)\sqcup B \sqcup A$ defined by $\tilde{g}_0 = g_0 = \iota _2$ and $\tilde{g}_1 = -g_1$ is the inverse of *g* in the sense that $g\bullet _{X_B\times D} \tilde{g} = \tilde{g}\bullet _{X_B\times D} g = e_{X_B\times D}$.

### Proof

From the hypothesis, (6.2) and $x^2=0$, it follows that

7.12 $$\begin{aligned} (g\bullet _{X_B\times D} \tilde{g})\big (a\big )&= \mathbbm {1}_{F_{{\varvec{0}}}/(x^2)}\otimes \mathbbm {1}_{B} \otimes a + x\otimes \big (g_{1}(a) + \tilde{g}_1(a)\big ) \nonumber \\&= \mathbbm {1}_{F_{{\varvec{0}}}/(x^2)}\otimes \mathbbm {1}_{B} \otimes a = e_{X_B\times D}(a), \end{aligned}$$

for all $a\in A$ and similarly that $\tilde{g}\bullet _{X_B\times D} g = e_{X_B\times D}$. $\square$

This result allows us to give a very explicit characterization of the functor underlying the object $T_e\mathrm {Aut}(X_A)$ in ${^{H}}\mathscr {G}$. Let us define the functor ${^{H}}\mathrm {Der}(A,-\sqcup A){:}\,{^{H}}\mathscr {S}^\mathrm {op}\rightarrow \mathsf {Set}$ on objects $X_B$ by

7.13a $$\begin{aligned} {^{H}}\mathrm {Der}(A,-\sqcup A)\big (X_B\big ) := {^{H}}\mathrm {Der}(A,B\sqcup A), \end{aligned}$$

which is the subset of $v\in \mathrm {Hom}_{{^{H}}\mathscr {M}}(A,B\sqcup A)$ satisfying (7.11), and on morphisms $f{:}\,X_B\rightarrow X_{B^\prime }$ by

7.13b $$\begin{aligned} {^{H}}\mathrm {Der}(A,-\sqcup A)\big (f\big ){:}\, {^{H}}\mathrm {Der}(A,B^\prime \sqcup A)&\longrightarrow {^{H}}\mathrm {Der}(A,B\sqcup A),\nonumber \\ \big (v{:}\,A\rightarrow B^\prime \sqcup A\big )&\longmapsto \big ((f^*\otimes \mathrm {id}_{A})\circ v{:}\,A\rightarrow B \sqcup A\big ). \end{aligned}$$

### Corollary 7.5

The presheaf underlying $T_e\mathrm {Aut}(X_A)$ is isomorphic to ${^{H}}\mathrm {Der}(A,-\sqcup A)$ via the natural isomorphism with components

7.14 $$\begin{aligned} T_e\mathrm {Aut}(X_A)\big (X_B\big )\longrightarrow {^{H}}\mathrm {Der}(A,B\sqcup A),~~g\longmapsto g_1, \end{aligned}$$

where $g_1$ is defined according to (7.9). Hence, ${^{H}}\mathrm {Der}(A,-\sqcup A)$ is a sheaf, i.e., an object in ${^{H}}\mathscr {G}$, and $T_e\mathrm {Aut}(X_A)$ is also isomorphic to ${^{H}}\mathrm {Der}(A,-\sqcup A)$ in ${^{H}}\mathscr {G}$.

### Proof

Since *g* is uniquely specified by $g_0,g_1$ [via (7.9)] and $g_0 = \iota _2$ for all $g\in T_e\mathrm {Aut}(X_A)(X_B)$, it follows that (7.14) is injective. Surjectivity of (7.14) follows from Lemma 7.4. Naturality of (7.14) is obvious. Because $T_e\mathrm {Aut}(X_A)$ and ${^{H}}\mathrm {Der}(A,-\sqcup A)$ are isomorphic as presheaves and $T_e\mathrm {Aut}(X_A)$ is a sheaf, it follows from the fully faithful embedding $\mathrm {Sh}({^{H}}\mathscr {S})\rightarrow \mathrm {PSh}({^{H}}\mathscr {S})$ that ${^{H}}\mathrm {Der}(A,-\sqcup A)$ is a sheaf and that the isomorphism is in ${^{H}}\mathscr {G}$. $\square$

We conclude by showing that ${^{H}}\mathrm {Der}(A,-\sqcup A)$ [and hence by Corollary 7.5 also $T_e\mathrm {Aut}(X_A)$] is a $\underline{K}$-module object in ${^{H}}\mathscr {G}$ that can be equipped with a Lie bracket. From the perspective used in Remark 7.1, this is equivalent to equipping ${^{H}}\mathrm {Der}(A,B\sqcup A)$ with a $B^{{\varvec{0}}}$-module structure and a Lie bracket on this $B^{{\varvec{0}}}$-module, such that both structures are natural transformations for ${^{H}}\mathscr {S}$-morphisms $f{:}\,X_{B}\rightarrow X_{B^\prime }$. Recall that ${^{H}}\mathrm {Der}(A,B\sqcup A)$ is the subset of $\mathrm {Hom}_{{^{H}}\mathscr {M}}(A,B\sqcup A)$ specified by the Leibniz rule (7.11). Because the Leibniz rule is a linear condition, it follows that ${^{H}}\mathrm {Der}(A,B\sqcup A)$ is closed under taking sums and additive inverses, and that it contains the zero map. From (7.13), one immediately sees that this Abelian group structure is natural with respect to ${^{H}}\mathscr {S}$-morphisms $f{:}\,X_{B}\rightarrow X_{B^\prime }$; hence, ${^{H}}\mathrm {Der}(A,-\sqcup A)$ is an Abelian group object in ${^{H}}\mathscr {G}$. The $B^{{\varvec{0}}}$-module structure

7.15a $$\begin{aligned} B^{{\varvec{0}}}\times {^{H}}\mathrm {Der}(A,B\sqcup A)\longrightarrow {^{H}}\mathrm {Der}(A,B\sqcup A),~~(b,v)\longmapsto b\cdot v \end{aligned}$$

is defined by setting

7.15b $$\begin{aligned} (b\cdot v) (a) := (b\otimes \mathbbm {1}_A)~v(a), \end{aligned}$$

for all $a\in A$. In order to verify that $(b\cdot v)\in {^{H}}\mathrm {Der}(A,B\sqcup A)$, i.e., that it is *H*-equivariant and satisfies the Leibniz rule (7.11), it is essential to use the fact that *b* is coinvariant, $\rho ^B{:}\,b\mapsto \mathbbm {1}_H\otimes b$. From (7.13), one immediately sees that this $B^{{\varvec{0}}}$-module structure is natural with respect to ${^{H}}\mathscr {S}$-morphisms $f{:}\,X_{B}\rightarrow X_{B^\prime }$, i.e.,

7.16 $$\begin{aligned} (f^*\otimes \mathrm {id}_{A})\circ (b^\prime \cdot v^\prime ) = f^*(b^\prime )\cdot \big ((f^*\otimes \mathrm {id}_{A})\circ v^\prime \, \big ), \end{aligned}$$

for all $b^\prime \in {B^\prime }\,^{{\varvec{0}}}$ and $v^\prime \in {^{H}}\mathrm {Der}(A,B^\prime \sqcup A)$. This endows ${^{H}}\mathrm {Der}(A,-\sqcup A)$ with the structure of a $\underline{K}$-module object in ${^{H}}\mathscr {G}$.

It remains to define a Lie bracket

7.17a $$\begin{aligned}{}[-,-]_{X_B}^{}{:}\, {^{H}}\mathrm {Der}(A,B\sqcup A)\otimes _{B^{{\varvec{0}}}}{^{H}}\mathrm {Der}(A,B\sqcup A)\longrightarrow {^{H}}\mathrm {Der}(A,B\sqcup A) \end{aligned}$$

on each $B^{{\varvec{0}}}$-module ${^{H}}\mathrm {Der}(A,B\sqcup A)$. Let us set

7.17b $$\begin{aligned}{}[v,w]_{X_B}^{} := (\mu _B\otimes \mathrm {id}_A)\circ \big ( (\mathrm {id}_B\otimes v)\circ w - (\mathrm {id}_B\otimes w)\circ v\big ), \end{aligned}$$

for all $v,w\in {^{H}}\mathrm {Der}(A,B\sqcup A)$, where $\mu _B{:}\,B\otimes B\rightarrow B$ is the product on *B*. Notice that $[v,w]_{X_B}{:}\,A\rightarrow B\sqcup A$ is an ${^{H}}\mathscr {M}$-morphism. A straightforward but slightly lengthy computation (using the Leibniz rule (7.11) for *v* and *w*) shows that $[v,w]_{X_B}$ satisfies the Leibniz rule; hence, it is an element in ${^{H}}\mathrm {Der}(A,B\sqcup A)$. Antisymmetry of $[\,-,\,-\,]_{X_B}$ follows immediately from the definition, and the Jacobi identity is shown by direct computation. Moreover, $B^{{\varvec{0}}}$-linearity of the Lie bracket, i.e.,

7.18 $$\begin{aligned}{}[b\cdot v,w]_{X_B} = b\cdot [v,w]_{X_B}= [v,b\cdot w]_{X_B}, \end{aligned}$$

for all $b\in B^{{\varvec{0}}}$ and $v,w\in {^{H}}\mathrm {Der}(A,B\sqcup A)$, can be easily verified by using the fact that *b* is coinvariant, and hence, it commutes with any other element in *B* [cf. (3.3)]. Naturality of the Lie bracket with respect to ${^{H}}\mathscr {S}$-morphisms $f{:}\,X_{B}\rightarrow X_{B^\prime }$ is a simple consequence of the fact that $f^*{:}\,B^\prime \rightarrow B$ preserves the products entering the definition in (7.17). We have therefore obtained an explicit description of the Lie algebra of the automorphism group $\mathrm {Aut}(X_A)$.

### Proposition 7.6

The functor ${^{H}}\mathrm {Der}(A,-\sqcup A)$ equipped with the structure morphisms introduced above is a Lie algebra object in the category $\mathrm {Mod}_{\underline{K}}({^{H}}\mathscr {G})$ of $\underline{K}$-module objects in ${^{H}}\mathscr {G}$.

## Braided derivations

The Lie algebra object ${^{H}}\mathrm {Der}(A,-\sqcup A)$ constructed in Proposition 7.6 is (isomorphic to) the Lie algebra of the automorphism group $\mathrm {Aut}(X_A)$. Hence, we may interpret it as the Lie algebra of infinitesimal automorphisms of the toric noncommutative space $X_A$ with function ${^{H}}\mathscr {A}_{\mathrm {fp}}$-algebra *A*. Another (a priori unrelated) way to think about the infinitesimal automorphisms of $X_A$ is to consider the Lie algebra $\mathrm {der}(A)$ of braided derivations of *A*, see [2–6]. In this section, we show that these two points of view are equivalent.

We briefly introduce the concept of braided derivations of ${^{H}}\mathscr {A}_{\mathrm {fp}}$-algebras *A*. Let us first consider the case where $A= F_{{\varvec{m}}_1,\ldots ,{\varvec{m}}_{N}}$ is the free ${^{H}}\mathscr {A}$-algebra with *N* generators $x_i$ with left *H*-coaction $x_i\mapsto t_{{\varvec{m}}_i}\otimes x_i$, for $i=1,\ldots ,N$. Let $\partial _j{:}\,F_{{\varvec{m}}_1,\ldots ,{\varvec{m}}_{N}} \rightarrow F_{{\varvec{m}}_1,\ldots ,{\varvec{m}}_{N}}$, for $j=1,\ldots ,N$, be the linear map defined by

8.1a $$\begin{aligned} \partial _j(x_i)&= \delta _{ij}\, \mathbbm {1},\end{aligned}$$

8.1b $$\begin{aligned} \partial _j(a\,a^\prime )&= \partial _j(a)\,a^\prime + R(a_{(-1)}\otimes t_{-{\varvec{m}}_j}) ~a_{(0)} \,\partial _j(a^\prime ), \end{aligned}$$

for all $i=1,\ldots ,N$ and $a,a^\prime \in F_{{\varvec{m}}_1,\ldots ,{\varvec{m}}_{N}}$. The map $\partial _j$ should be interpreted as the ‘partial derivative’ along the generator $x_j$; hence, it is natural to assign to it the left *H*-coaction $\partial _j \mapsto t_{-{\varvec{m}}_j}\otimes \partial _j$. It satisfies a braided generalization of the Leibniz rule that is controlled by the cotriangular structure *R*. Let us define the left *H*-comodule

8.2 $$\begin{aligned} \mathrm {der}\left( F_{{\varvec{m}}_1,\ldots ,{\varvec{m}}_N}\right) := \coprod _{j=1,\ldots ,N} \, F_{{\varvec{m}}_1,\ldots ,{\varvec{m}}_N}[-{\varvec{m}}_j], \end{aligned}$$

with the coproduct taken in ${^{H}}\mathscr {M}$, where for an object *V* in ${^{H}}\mathscr {M}$ we denote by $V[{\varvec{m}}]$ the object in ${^{H}}\mathscr {M}$ which has the same underlying vector space as *V* but which is equipped with the shifted left *H*-coaction

8.3 $$\begin{aligned} \rho ^{V[{\varvec{m}}]}{:}\,V\longrightarrow H\otimes V,~~v\longmapsto v_{(-1)}\,t_{{\varvec{m}}} \otimes v_{(0)}. \end{aligned}$$

We denote elements $L \in \mathrm {der}(F_{{\varvec{m}}_1,\ldots ,{\varvec{m}}_N})$ by $L=\sum _j\, L_j\,\partial _j$, where $L_j\in F_{{\varvec{m}}_1,\ldots ,{\varvec{m}}_N}$, because the *H*-coaction then takes the convenient form $\rho ^{\mathrm {der}(F_{{\varvec{m}}_1,\ldots ,{\varvec{m}}_N})}(L) =\sum _j\, {L_j}_{(-1)}\,t_{-{\varvec{m}}_j} \otimes {L_{j}}_{(0)} \,\partial _j$. The evaluation of $\mathrm {der}(F_{{\varvec{m}}_1,\ldots ,{\varvec{m}}_N})$ on $F_{{\varvec{m}}_1,\ldots ,{\varvec{m}}_N}$ is given by the ${^{H}}\mathscr {M}$-morphism

8.4 $$\begin{aligned} \mathrm {ev}{:}\,\mathrm {der}(F_{{\varvec{m}}_1,\ldots ,{\varvec{m}}_N}) \otimes F_{{\varvec{m}}_1,\ldots ,{\varvec{m}}_N}\longrightarrow F_{{\varvec{m}}_1,\ldots ,{\varvec{m}}_N},~~L\otimes a \longmapsto \sum _{j}\, L_j\, \partial _j(a). \end{aligned}$$

It is then easy to confirm the braided Leibniz rule

8.5 $$\begin{aligned} \mathrm {ev}\big (L\otimes (a\,a^\prime )\big ) = \mathrm {ev}(L\otimes a)\,a^\prime + R\left( a_{(-1)}\otimes L_{(-1)}\right) ~a_{(0)} \,\mathrm {ev}\left( L_{(0)}\otimes a^\prime \right) , \end{aligned}$$

for all $L\in \mathrm {der}(F_{{\varvec{m}}_1,\ldots ,{\varvec{m}}_N})$ and $a,a^\prime \in F_{{\varvec{m}}_1,\ldots ,{\varvec{m}}_N}$, which allows us to interpret elements of $\mathrm {der}(F_{{\varvec{m}}_1,\ldots ,{\varvec{m}}_N})$ as braided derivations.

For a finitely presented ${^{H}}\mathscr {A}$-algebra $A = F_{{\varvec{m}}_1,\ldots ,{\varvec{m}}_N}{/}(f_k)$, the left *H*-comodule of braided derivations is defined by

8.6 $$\begin{aligned} \mathrm {der}(A) := \left\{ L \in {\coprod \limits _{j=1,\ldots ,N}}\, A[-{\varvec{m}}_j]{:}\, {\sum \limits _j}\, L_j\,\partial _j(f_k)= 0~~\forall k\, \right\} \ \subseteq \ \coprod _{j=1,\ldots ,N}\, A[-{\varvec{m}}_j]. \end{aligned}$$

The evaluation ${^{H}}\mathscr {M}$-morphism is similar to that in the case of free ${^{H}}\mathscr {A}$-algebras and is given by

8.7 $$\begin{aligned} \mathrm {ev}{:}\,\mathrm {der}(A) \otimes A \longrightarrow A,~~L\otimes a \longmapsto \sum _{j}\, L_j\, \partial _j(a). \end{aligned}$$

Notice that $\mathrm {ev}$ is well defined because of the conditions imposed in (8.6). The braided Leibniz rule (8.5) also holds in the case of finitely presented ${^{H}}\mathscr {A}$-algebras.

### Proposition 8.1

Let *A* be any object in ${^{H}}\mathscr {A}_{\mathrm {fp}}$. Then, $\mathrm {der}(A)$ is a Lie algebra object in ${^{H}}\mathscr {M}$ with Lie bracket ${^{H}}\mathscr {M}$-morphism $[-,\,-]{:}\,\mathrm {der}(A)\otimes \mathrm {der}(A) \rightarrow \mathrm {der}(A)$ uniquely defined by

8.8 $$\begin{aligned} \mathrm {ev}\big ([L,L^\prime ]\otimes a\big ) := \mathrm {ev}\big (L\otimes \mathrm {ev}(L^\prime \otimes a)\big ) - R(L^\prime _{(-1)}\otimes L_{(-1)})~\mathrm {ev}\big (L^\prime _{(0)}\otimes \mathrm {ev}(L_{(0)}\otimes a)\big ), \end{aligned}$$

for all $L,L^\prime \in \mathrm {der}(A)$ and $a\in A$.

### Proof

Using the braided Leibniz rule (8.5), we can compute the right-hand side of (8.8) and obtain

8.9 $$\begin{aligned} \mathrm {ev}\big ([L,L^\prime ]\otimes a\big ) = \sum \limits _{j,k} \, \left( L_j\,\partial _j(L^\prime _k) -R({L^\prime _j}_{(-1)}\,t_{-{\varvec{m}}_j}\otimes {L_k}_{(-1)}\,\,t_{-{\varvec{m}}_k})~{L^\prime _j}_{(0)}\,\partial _j({L_k}_{(0)})\right) \, \partial _k(a). \end{aligned}$$

Hence, $[L,L^\prime \,]$ is uniquely defined and it is a braided derivation. The braided antisymmetry and Jacobi identity on $[-,\,-]$ can be verified by a straightforward computation. $\square$

We would now like to compare the braided derivations $\mathrm {der}(A)$ with the automorphism Lie algebra ${^{H}}\mathrm {Der}(A,-\sqcup A)$ constructed in Sect. 7. There is, however, a problem: While $\mathrm {der}(A)$ is a Lie algebra object in the category ${^{H}}\mathscr {M}$ of left *H*-comodules, ${^{H}}\mathrm {Der}(A,-\sqcup A)$ is a Lie algebra object in the category $\mathrm {Mod}_{\underline{K}}({^{H}}\mathscr {G})$ of $\underline{K}$-module objects in the category ${^{H}}\mathscr {G}$ of generalized toric noncommutative spaces. We show in “Appendix” that there exists a functor $j{:}\,{^{H}}\mathscr {M}\rightarrow \mathrm {Mod}_{\underline{K}}({^{H}}\mathscr {G})$, which becomes a fully faithful embedding when restricted to the full subcategory ${^{H}}\mathscr {M}_{\mathrm {dec}}$ of decomposable left *H*-comodules (cf. Definition 9.1). Because $\mathrm {der}(A)$ is a decomposable left *H*-comodule (cf. Corollary 9.4), we may use the fully faithful embedding $j{:}\,{^{H}}\mathscr {M}_{\mathrm {dec}} \rightarrow \mathrm {Mod}_{\underline{K}}({^{H}}\mathscr {G})$ to relate $\mathrm {der}(A)$ to ${^{H}}\mathrm {Der}(A,-\sqcup A)$.

Let us characterize more explicitly the object $j(\mathrm {der}(A))$ in $\mathrm {Mod}_{\underline{K}}({^{H}}\mathscr {G})$. Its underlying functor [cf. (9.7)] assigns to an object $X_B$ in ${^{H}}\mathscr {S}$ the $B^{\varvec{0}}$-module

8.10a $$\begin{aligned} j(\mathrm {der}(A))(X_B) = \big (B\otimes \mathrm {der}(A)\big )^{{\varvec{0}}} \end{aligned}$$

and to an ${^{H}}\mathscr {S}$-morphism $f{:}\,X_B\rightarrow X_C$ the module morphism

8.10b $$\begin{aligned} j(\mathrm {der}(A))(f) = (f^*\otimes \mathrm {id}_{\mathrm {der}(A)}){:}\, \big (C\otimes \mathrm {der}(A)\big )^{{\varvec{0}}}\longrightarrow \big (B\otimes \mathrm {der}(A)\big )^{{\varvec{0}}}. \end{aligned}$$

For any object $X_B$ in ${^{H}}\mathscr {S}$, we define a map

8.11a $$\begin{aligned} \xi _{X_B}{:}\,\big (B\otimes \mathrm {der}(A)\big )^{{\varvec{0}}} \longrightarrow {^{H}}\mathrm {Der}(A,B\sqcup A),~~ b\otimes L \longmapsto \xi _{X_B} (b\otimes L) \end{aligned}$$

by setting

8.11b $$\begin{aligned} \xi _{X_B}(b\otimes L)(a) := b\otimes \mathrm {ev}(L\otimes a) = b\otimes \left( \sum _j\, L_j\,\partial _j(a)\right) , \end{aligned}$$

for all $a\in A$. It is easy to check that $\xi _{X_B}(b\otimes L){:}\,A\rightarrow B\sqcup A$ is an ${^{H}}\mathscr {M}$-morphism by using the property that $b\otimes L$ is *H*-coinvariant. Moreover, $\xi _{X_B}(b\otimes L)$ satisfies the Leibniz rule (7.11) because *L* satisfies the braided Leibniz rule (8.5) and $b\otimes L$ is *H*-coinvariant. Explicitly we have

8.12 $$\begin{aligned} \xi _{X_B}(b\otimes L)(a\,a^\prime )&= b\otimes \left( \mathrm {ev}(L\otimes a)\,a^\prime + R(a_{(-1)}\otimes L_{(-1)})\, a_{(0)}\, \mathrm {ev}(L_{(0)}\otimes a^\prime )\right) \nonumber \\&=\xi _{X_B}(b\otimes L)(a)\,(\mathbbm {1}_B\otimes a^\prime ) + (\mathbbm {1}_B\otimes a)\, \xi _{X_B}(b\otimes L)(a^\prime ), \end{aligned}$$

where the last step follows from (3.19) and (2.4). This shows that the image of $\xi _{X_B}$ lies in ${^{H}}\mathrm {Der}(A,B\sqcup A)$, as we have asserted in (8.11).

The maps $\xi _{X_B}$ are clearly $B^{{\varvec{0}}}$-module morphisms with respect to the $B^{\varvec{0}}$-module structure on ${^{H}}\mathrm {Der}(A,B\sqcup A)$ introduced in (7.15) and that on $j(\mathrm {der}(A))(X_B)$ introduced in (9.10), and they are natural with respect to ${^{H}}\mathscr {S}$-morphisms $f{:}\,X_B\rightarrow X_C$. Hence, we have defined a morphism

8.13 $$\begin{aligned} \xi {:}\, j(\mathrm {der}(A)) \longrightarrow {^{H}}\mathrm {Der}(A,-\sqcup A)~ \end{aligned}$$

in the category $\mathrm {Mod}_{\underline{K}}({^{H}}\mathscr {G})$ of $\underline{K}$-module objects in ${^{H}}\mathscr {G}$. The main result of this section is

### Theorem 8.2

The $\mathrm {Mod}_{\underline{K}}({^{H}}\mathscr {G})$-morphism (8.13) is an isomorphism. Hence, $\mathrm {der}(A)$ and ${^{H}}\mathrm {Der}(A,-\sqcup A)$ are equivalent descriptions of the infinitesimal automorphisms of a toric noncommutative space $X_A$.

### Proof

Fix a presentation $A = F_{{\varvec{m}}_1,\ldots ,{\varvec{m}}_N}/(f_k)$ of *A* and any object $X_B$ in ${^{H}}\mathscr {S}$. We define *nonequivariant* linear maps $\widehat{\partial }_j{:}\,F_{{\varvec{m}}_1,\ldots ,{\varvec{m}}_N} \rightarrow B\otimes F_{{\varvec{m}}_1,\ldots ,{\varvec{m}}_N}$ by setting

8.14a $$\begin{aligned} \widehat{\partial }_j(x_i)&=\delta _{ij}~ \mathbbm {1}_B\otimes \mathbbm {1}_{F_{{\varvec{m}}_1,\ldots ,{\varvec{m}}_N}}, \end{aligned}$$

8.14b $$\begin{aligned} \widehat{\partial }_j(a\,a^\prime )&= \widehat{\partial }_j(a)\, (\mathbbm {1}_B\otimes a) + R(a_{(-1)}\otimes t_{-{\varvec{m}}_j})~(\mathbbm {1}_B\otimes a_{(0)}) \, \widehat{\partial }_j(a^\prime ), \end{aligned}$$

for all generators $x_i$ and all $a,a^\prime \in F_{{\varvec{m}}_1,\ldots ,{\varvec{m}}_N}$. There is an isomorphism

8.15 $$\begin{aligned} \left\{ v\in {\coprod \limits _{j=1,\ldots ,N}\, (B\otimes A[-{\varvec{m}}_j])^{{\varvec{0}}}{:}\,\sum \limits _j\, v_j \, \widehat{\partial }_j(f_k) =0~~\forall k}\right\} \simeq {^{H}}\mathrm {Der}(A,B\sqcup A) \end{aligned}$$

given by the assignment $v \mapsto \sum _j\, v_j\,\widehat{\partial }_j$. Because *A* and *B* are decomposable, we obtain a chain of isomorphisms

8.16 $$\begin{aligned} \coprod _{j=1,\ldots ,N} (B\otimes A[-{\varvec{m}}_j])^{{\varvec{0}}}&\simeq \coprod _{j=1,\ldots ,N} \ \coprod _{{\varvec{n}}\in \mathbb {Z}^n} \, \big (B^{{\varvec{n}}}\otimes A[-{\varvec{m}}_j]^{-{\varvec{n}}}\big ) \nonumber \\&\simeq \coprod _{{\varvec{n}}\in \mathbb {Z}^n} \, \left( B^{{\varvec{n}}}\otimes \left( \coprod _{j=1,\ldots ,N} \, A[-{\varvec{m}}_j]\right) ^{-{\varvec{n}}}\right) \nonumber \\&\simeq \left( B\otimes \coprod _{j=1,\ldots ,N}\, A[-{\varvec{m}}_j]\right) ^{{\varvec{0}}}. \end{aligned}$$

The resulting isomorphism preserves the conditions imposed in (8.15) and (8.6); hence, it induces an isomorphism between ${^{H}}\mathrm {Der}(A,B\sqcup A)$ and $j(\mathrm {der}(A))(X_B)$. $\square$

### Remark 8.3

Even though the functor $j{:}\,{^{H}}\mathscr {M}\rightarrow \mathrm {Mod}_{\underline{K}}({^{H}}\mathscr {G})$ is not monoidal (cf. Remark 9.7), there exists a $\mathrm {Mod}_{\underline{K}}({^{H}}\mathscr {G})$-morphism

8.17 $$\begin{aligned} \psi {:}\,j(\mathrm {der}(A))\otimes j(\mathrm {der}(A)) \longrightarrow j(\mathrm {der}(A)\otimes \mathrm {der}(A)), \end{aligned}$$

which is described explicitly in (9.13). We now confirm that the isomorphism $\xi {:}\,j(\mathrm {der}(A)) \rightarrow {^{H}}\mathrm {Der}(A,-\sqcup A)$ established in Theorem 8.2 preserves the Lie brackets on $\mathrm {der}(A)$ and ${^{H}}\mathrm {Der}(A,-\sqcup A)$ in the sense that the diagram8.18in $\mathrm {Mod}_{\underline{K}}({^{H}}\mathscr {G})$ commutes. Fixing an arbitrary object $X_B\in {^{H}}\mathscr {S}$ and going along the upper path of this diagram, we obtain

8.19a $$\begin{aligned}&\left( \xi _{X_B}\circ (\mathrm {id}_{B}\otimes [\,-,\,-\,])\circ \psi _{X_B}\big ((b\otimes L) \otimes _{B^{{\varvec{0}}}} (b^\prime \otimes L^\prime )\big )\right) (a)\nonumber \\&\quad =R(b^\prime _{(-1)}\otimes L_{(-1)}) ~b\,b^\prime _{(0)}\otimes \mathrm {ev}\big ([L_{(0)},L^\prime ]\otimes a\big )\nonumber \\&\quad = R(b^\prime _{{(-1)}_{(1)}}\otimes L_{(-1)}) ~R(b^\prime _{{(-1)}_{(2)}}\otimes b_{(-1)})~b^\prime _{(0)}\,b_{(0)} \otimes \mathrm {ev}\big ([L_{(0)},L^\prime ]\otimes a\big )\nonumber \\&\quad = R(b^\prime _{{(-1)}}\otimes b_{(-1)}\, L_{(-1)}) ~b^\prime _{(0)}\,b_{(0)} \otimes \mathrm {ev}\big ([L_{(0)},L^\prime ]\otimes a\big )\nonumber \\&\quad = b^\prime \,b\otimes \mathrm {ev}\big ([L,L^\prime ]\otimes a\big ), \end{aligned}$$

for all $a\in A$, where in the last two steps we used the properties (2.4) of the cotriangular structure *R* and the fact that $b\otimes L \in (B\otimes \mathrm {der}(A))^{{\varvec{0}}}$ is coinvariant. Going now along the lower path of the diagram, we obtain

8.19b $$\begin{aligned} \big [\xi _{X_B}(b\otimes L), \xi _{X_B}(b^\prime \otimes L^\prime )\big ]_{X_B}(a)= b^\prime \,b\otimes \mathrm {ev}\big (L\otimes \mathrm {ev}(L^\prime \otimes a)\big ) - b\,b^\prime \otimes \mathrm {ev}\big (L^\prime \otimes \mathrm {ev}(L\otimes a)\big ), \end{aligned}$$

for all $a\in A$, where we used the definition of the Lie bracket $[-,\,-]_{X_B}$ given in (7.17). These two expressions coincide because, using without loss of generality $b\otimes L\in B^{{\varvec{m}}}\otimes \mathrm {der}(A)^{-{\varvec{m}}}$ and $b^\prime \otimes L^\prime \in B^{{\varvec{m}}^\prime }\otimes \mathrm {der}(A)^{-{\varvec{m}}^\prime }$, the second term in (8.19b) can be rearranged as

8.20 $$\begin{aligned} b\,b^\prime \otimes \mathrm {ev}\big (L^\prime \otimes \mathrm {ev}(L\otimes a)\big )&= R(b^\prime _{(-1)}\otimes b_{(-1)})~ b^{\prime }_{(0)}\,b_{(0)} \otimes \mathrm {ev}\big (L^\prime \otimes \mathrm {ev}(L\otimes a)\big )\nonumber \\&= R(t_{{\varvec{m}}^\prime }\otimes t_{{\varvec{m}}})~ b^{\prime }\,b \otimes \mathrm {ev}\big (L^\prime \otimes \mathrm {ev}(L\otimes a)\big )\nonumber \\&= R(t_{-{\varvec{m}}^\prime }\otimes t_{-{\varvec{m}}})~ b^{\prime }\,b \otimes \mathrm {ev}\big (L^\prime \otimes \mathrm {ev}(L\otimes a)\big )\nonumber \\&= R(L_{(-1)}^\prime \otimes L_{(-1)})~ b^{\prime }\,b \otimes \mathrm {ev}\big (L_{(0)}^\prime \otimes \mathrm {ev}(L_{(0)}\otimes a)\big ), \end{aligned}$$

for all $a\in A$, where in the third step we used the property $R(h\otimes g) = R(S(h)\otimes S(g))$, for all $h,g\in H$, see, e.g., [23, Lemma 2.2.2].

## Appendix: Technical details for Sect. 8

### Decomposable objects in ${^{H}}\mathscr {M}$

Given any object *V* in ${^{H}}\mathscr {M}$, we define

9.1 $$\begin{aligned} V^{{\varvec{m}}} := \big \{ v\in V{:}\, \rho ^V(v) = t_{{\varvec{m}}}\otimes v\big \}, \end{aligned}$$

for all ${\varvec{m}}\in \mathbb {Z}^n$. Notice that $V^{{\varvec{0}}}$ is the vector space of coinvariants and that $V^{{\varvec{m}}} \subseteq V$ are ${^{H}}\mathscr {M}$-subobjects, for all ${\varvec{m}}$.

#### Definition 9.1

An object *V* in ${^{H}}\mathscr {M}$ is decomposable if the canonical ${^{H}}\mathscr {M}$-morphism

9.2 $$\begin{aligned} \coprod _{{\varvec{m}}\in \mathbb {Z}^n}\, V^{{\varvec{m}}} \longrightarrow V,~~\coprod _{{\varvec{m}}}\, v_{{\varvec{m}}} \longmapsto \sum _{{\varvec{m}}}\, v_{{\varvec{m}}} \end{aligned}$$

is an isomorphism. We denote by ${^{H}}\mathscr {M}_{\mathrm {dec}}$ the full subcategory of decomposables.

#### Lemma 9.2

(Properties of decomposables) 

- Tensor products of decomposables are decomposable, i.e., ${^{H}}\mathscr {M}_{\mathrm {dec}}$ is a monoidal subcategory of ${^{H}}\mathscr {M}$.
- Coproducts of decomposables are decomposable.
- ${^{H}}\mathscr {M}$-subobjects of decomposables are decomposable.

#### Proof

To prove item (a), note that for *V*, *W* decomposable we have

9.3 $$\begin{aligned} V\otimes W \simeq \coprod _{{\varvec{m}}\in \mathbb {Z}^{n}} \, \left( \coprod _{{\varvec{n}}\in \mathbb {Z}^n} \, \left( V^{{\varvec{n}}}\otimes W^{{\varvec{m}}-{\varvec{n}}}\right) \right) ; \end{aligned}$$

hence, $V\otimes W$ is decomposable with

9.4 $$\begin{aligned} (V\otimes W)^{{\varvec{m}}} = \coprod _{{\varvec{n}}\in \mathbb {Z}^n}\, \big (V^{{\varvec{n}}}\otimes W^{{\varvec{m}}-{\varvec{n}}}\big ). \end{aligned}$$

The monoidal unit object $\mathbb {K}_{{\varvec{0}}}$ is clearly decomposable. Items (b) and (c) are obvious. $\square$

#### Lemma 9.3

Let *A* be an object in ${^{H}}\mathscr {A}_{\mathrm {fp}}$. Then, the left *H*-comodule underlying *A* is decomposable.

#### Proof

Let us start with the case where $A = F_{{\varvec{m_1}},\ldots ,{\varvec{m}}_N}$ is a free ${^{H}}\mathscr {A}$-algebra. As $F_{{\varvec{m_1}},\ldots ,{\varvec{m}}_N} \simeq F_{{\varvec{m}}_1} \sqcup \cdots \sqcup F_{{\varvec{m}}_M}$, where $\sqcup$ denotes the coproduct in ${^{H}}\mathscr {A}_{\mathrm {fp}}$ (given explicitly by $\otimes$), we can use Lemma 9.2(a) and reduce the problem to show that $F_{{\varvec{m}}}$ is decomposable. Notice that

9.5 $$\begin{aligned} {F_{{\varvec{m}}}}^{{\varvec{n}}} = {\left\{ \begin{array}{ll} \mathrm {span}_{\mathbb {K}}(x^k)\simeq \mathbb {K}_{{\varvec{n}}}, &{}~~\text {for }{\varvec{n}} = k\,{\varvec{m}},~k\in \mathbb {Z}_{\ge 0},\\ 0, &{}~~\text {otherwise}, \end{array}\right. } \end{aligned}$$

where *x* denotes the generator of $F_{{\varvec{m}}}$ with *H*-coaction $x\mapsto t_{{\varvec{m}}}\otimes x$. The canonical ${^{H}}\mathscr {M}$-morphism reads as

9.6 $$\begin{aligned} \coprod _{{\varvec{n}}\in \mathbb {Z}^n}\, {F_{{\varvec{m}}}}^{{\varvec{n}}} \simeq \coprod _{k\in \mathbb {Z}_{\ge 0}}\, \mathbb {K}_{k\,{\varvec{m}}}\longrightarrow F_{{\varvec{m}}},~~\coprod _{k}\, c_k \longmapsto \sum _{k}\, c_k\,x^k, \end{aligned}$$

and it is easy to see that it is an isomorphism.

For the case where $A = F_{{\varvec{m}}_1,\ldots ,{\varvec{m}}_N}{/}I$ is finitely presented, we use the property that $F_{{\varvec{m}}_1,\ldots ,{\varvec{m}}_N}$ is decomposable and hence so is the ${^{H}}\mathscr {A}$-ideal $I\subseteq F_{{\varvec{m}}_1,\ldots ,{\varvec{m}}_N}$. Consequently, the quotient $A = F_{{\varvec{m}}_1,\ldots ,{\varvec{m}}_N}{/}I$ is decomposable as well. $\square$

#### Corollary 9.4

Let *A* be an object in ${^{H}}\mathscr {A}_{\mathrm {fp}}$. Then, $\mathrm {der}(A)$ is decomposable.

#### Proof

Recalling the definition of $\mathrm {der}(A)$ in (8.6), the claim follows from the fact that *A* is decomposable (cf. Lemma 9.3), and Lemma 9.2(b) and (c). $\square$

### Embedding of ${^{H}}\mathscr {M}$ into ${^{H}}\mathscr {G}$

We first define a functor

9.7a $$\begin{aligned} j{:}\,{^{H}}\mathscr {M}\longrightarrow \mathrm {PSh}({^{H}}\mathscr {S}). \end{aligned}$$

To an object *V* in ${^{H}}\mathscr {M}$, the functor *j* assigns the presheaf $j(V){:}\,{^{H}}\mathscr {S}^\mathrm {op}\rightarrow \mathsf {Set}$ that acts on objects $X_B$ as

9.7b $$\begin{aligned} j(V)(X_B) := (B\otimes V)^{{\varvec{0}}} \end{aligned}$$

and on morphisms $f{:}\,X_B\rightarrow X_C$ as

9.7c $$\begin{aligned} j(V)(f) := (f^*\otimes \mathrm {id}_V){:}\, (C\otimes V)^{{\varvec{0}}} \longrightarrow (B\otimes V)^{{\varvec{0}}}. \end{aligned}$$

To a morphism $L{:}\,V\rightarrow W$ in ${^{H}}\mathscr {M}$, the functor *j* assigns the presheaf morphism $j(L){:}\,j(V) \rightarrow j(W)$ given by the natural transformation with components

9.8 $$\begin{aligned} j(L)_{X_B} := (\mathrm {id}_{B}\otimes L){:}\, (B\otimes V)^{{\varvec{0}}} \longrightarrow (B\otimes W)^{{\varvec{0}}}. \end{aligned}$$

#### Proposition 9.5

For any object *V* in ${^{H}}\mathscr {M}$, the presheaf *j*(*V*) is a sheaf. Hence, (9.7) induces a functor $j{:}\,{^{H}}\mathscr {M}\rightarrow {^{H}}\mathscr {G}$.

#### Proof

Given any ${^{H}}\mathscr {S}$-Zariski covering family $\{f_i{:}\,X_{B[s_i^{-1}]} \rightarrow X_B^{}\}$, we have to verify the sheaf condition (5.2), i.e., that the diagram

9.9

is an equalizer in $\mathsf {Set}$. This follows from the same argument that we have used in the second paragraph of the proof of Proposition 5.1. $\square$

For any object $X_B$ in ${^{H}}\mathscr {S}$, the set $j(V)(X_B) = (B\otimes V)^{{\varvec{0}}}$ is a $B^{{\varvec{0}}}$-module with Abelian group structure induced by the vector space structure of $B\otimes V$ and $B^{{\varvec{0}}}$-action given by

9.10 $$\begin{aligned} B^{{\varvec{0}}} \times (B\otimes V)^{{\varvec{0}}}\longrightarrow (B\otimes V)^{{\varvec{0}}},~~(b,b^\prime \otimes v)\longmapsto b\cdot (b^\prime \otimes v) := (b\,b^\prime )\otimes v. \end{aligned}$$

These structures are natural with respect to ${^{H}}\mathscr {S}$-morphisms $f{:}\,X_{B}\rightarrow X_{C}$, i.e.,

9.11 $$\begin{aligned} (f^*\otimes \mathrm {id}_V) \big (c\cdot (c^\prime \otimes v)\big ) = f^*(c)\cdot \big ((f^*\otimes \mathrm {id}_V) (c^\prime \otimes v)\big ), \end{aligned}$$

for all $c\in C^{{\varvec{0}}}$, $c^\prime \in C$ and $v\in V$; hence, they endow *j*(*V*) with the structure of a $\underline{K}$-module object in ${^{H}}\mathscr {G}$. For any ${^{H}}\mathscr {M}$-morphism $L{:}\,V\rightarrow W$ the ${^{H}}\mathscr {G}$-morphism $j(L){:}\,j(V) \rightarrow j(W)$ is compatible with this $\underline{K}$-module object structure, i.e., (9.8) is a $B^{{\varvec{0}}}$-module morphism, for all objects $X_B$ in ${^{H}}\mathscr {S}$. We have therefore obtained

#### Proposition 9.6

With respect to the $\underline{K}$-module object structures on *j*(*V*) introduced above, $j{:}\,{^{H}}\mathscr {M}\rightarrow \mathrm {Mod}_{\underline{K}}({^{H}}\mathscr {G})$ is a functor with values in the category $\mathrm {Mod}_{\underline{K}}({^{H}}\mathscr {G})$ of $\underline{K}$-module objects in ${^{H}}\mathscr {G}$.

#### Remark 9.7

The functor *j* is not a monoidal functor, i.e., the object $j(V\otimes W)$ is in general not isomorphic to $j(V)\otimes j(W)$, where the tensor product in $\mathrm {Mod}_{\underline{K}}({^{H}}\mathscr {G})$ is given by

9.12 $$\begin{aligned} (j(V)\otimes j(W))(X_B) := j(V)(X_B)\otimes _{B^{\varvec{0}}} j(W)(X_B), \end{aligned}$$

for all objects $X_B$ in ${^{H}}\mathscr {S}$. For example, take $B=\mathbb {K}$ then $j(V\otimes W)(X_{\mathbb {K}}) = (V\otimes W)^{{\varvec{0}}}$ but $(j(V)\otimes j(W))(X_{\mathbb {K}}) = V^{\varvec{0}}\otimes W^{{\varvec{0}}}$. However, there exists a $\mathrm {Mod}_{\underline{K}}({^{H}}\mathscr {G})$-morphism

9.13a $$\begin{aligned} \psi {:}\,j(V)\otimes j(W) \longrightarrow j(V\otimes W), \end{aligned}$$

for all objects *V*, *W* in ${^{H}}\mathscr {M}$. The components of $\psi$ are given by

9.13b $$\begin{aligned} \psi _{X_B}{:}\,(B\otimes V)^{{\varvec{0}}}\otimes _{B^{{\varvec{0}}}} (B\otimes W)^{{\varvec{0}}}&\longrightarrow (B\otimes V\otimes W)^{{\varvec{0}}},\nonumber \\ (b\otimes v)\otimes _{B^{{\varvec{0}}}} (b^\prime \otimes w)&\longmapsto R(b^\prime _{(-1)}\otimes v_{(-1)}) ~ (b\,b^\prime _{(0)})\otimes v_{(0)}\otimes w, \end{aligned}$$

for all objects $X_B$ in ${^{H}}\mathscr {S}$.

Let now *V* be decomposable, i.e., an object in ${^{H}}\mathscr {M}_{\mathrm {dec}}$. Because any object *B* in ${^{H}}\mathscr {A}_{\mathrm {fp}}$ is decomposable as well (cf. Lemma 9.3), we obtain

9.14 $$\begin{aligned} j(V)(X_B) \simeq \coprod _{{\varvec{n}}\in \mathbb {Z}^n} \, \big (B^{{\varvec{n}}}\otimes V^{-{\varvec{n}}}\big ). \end{aligned}$$

For the special case where $B = F_{{\varvec{m}}}$ is the free ${^{H}}\mathscr {A}$-algebra with one generator with coaction $x\mapsto t_{{\varvec{m}}}\otimes x$, we use $B\simeq \coprod _{k\in \mathbb {Z}_{\ge 0}} \, \mathbb {K}_{k\,{\varvec{m}}}$ to simplify this expression further to

9.15 $$\begin{aligned} j(V)(X_{F_{{\varvec{m}}}}) \simeq \coprod _{k\in \mathbb {Z}_{\ge 0}} \, V^{-k\,{\varvec{m}}}, \end{aligned}$$

where the coproducts here are in the category of vector spaces. Using this explicit characterization, we can establish the main result of “Appendix.”

#### Theorem 9.8

For any two objects *V*, *W* in ${^{H}}\mathscr {M}_{\mathrm {dec}}$ there is a bijection of $\mathrm {Hom}$-sets

9.16 $$\begin{aligned} \mathrm {Hom}_{{^{H}}\mathscr {M}}(V,W) \simeq \mathrm {Hom}_{\mathrm {Mod}_{\underline{K}}({^{H}}\mathscr {G})} (j(V),j(W)). \end{aligned}$$

Thus, the restricted functor $j{:}\,{^{H}}\mathscr {M}_{\mathrm {dec}} \rightarrow \mathrm {Mod}_{\underline{K}}({^{H}}\mathscr {G})$ to the full subcategory of decomposables ${^{H}}\mathscr {M}_{\mathrm {dec}}$ is fully faithful.

#### Proof

Let $\eta {:}\,j(V)\rightarrow j(W)$ be any morphism in $\mathrm {Mod}_{\underline{K}}({^{H}}\mathscr {G})$. The components

9.17 $$\begin{aligned} \eta _{X_B}{:}\,(B\otimes V)^{{\varvec{0}}}\longrightarrow (B\otimes W)^{{\varvec{0}}}~ \end{aligned}$$

are $B^{{\varvec{0}}}$-module morphisms, for all objects $X_B$ in ${^{H}}\mathscr {S}$, such that for any ${^{H}}\mathscr {S}$-morphism $f{:}\,X_B\rightarrow X_C$ the diagram

9.18

commutes.

We first show that $\eta$ is uniquely determined by the components $\eta _{X_{F_{{\varvec{m}}}}}$, for all free ${^{H}}\mathscr {A}$-algebras $F_{{\varvec{m}}}$ with one generator. Using (9.14), we find that $\eta _{X_B}$ is specified by its action on elements of the form $b\otimes v \in B^{{\varvec{n}}}\otimes V^{-{\varvec{n}}}$, for all ${\varvec{n}}$. Given any such element, we define an ${^{H}}\mathscr {A}_{\mathrm {fp}}$-morphism $f^*{:}\,F_{{\varvec{n}}} \rightarrow B$ by sending $x\mapsto b$. (Notice that the morphism $f^*$ depends on the chosen element $b\otimes v$.) Then, the commutative diagram (9.18) implies that $\eta _{X_B}(b\otimes v) = (f^*\otimes \mathrm {id}_W)(\eta _{X_{F_{{\varvec{n}}}}}(x\otimes v))$; hence, the value of $\eta _{X_B}$ at $b\otimes v$ is fixed by $\eta _{X_{F_{{\varvec{n}}}}}$. As $b\otimes v$ was arbitrary, we find that $\eta$ is uniquely determined by the components $\{\eta _{X_{F_{{\varvec{m}}}}}{:}\,{\varvec{m}}\in \mathbb {Z}^n\}$.

In the next step, we show that the components $\{\eta _{X_{F_{{\varvec{m}}}}}{:}\,{\varvec{m}}\in \mathbb {Z}^n\}$ are uniquely determined by an ${^{H}}\mathscr {M}$-morphism $L{:}\,V\rightarrow W$. Consider the ${^{H}}\mathscr {A}_{\mathrm {fp}}$-morphism $f^*{:}\,F_{{\varvec{m}}}\rightarrow F_{{\varvec{m}}}$ defined by $x\mapsto c\,x$, where $c\in \mathbb {K}$ is an arbitrary constant. Using (9.15) and the commutative diagram (9.18) corresponding to this morphism, we obtain a commutative diagram

9.19

The vertical arrows map elements $v\in V^{-k\,{\varvec{m}}}$ to $c^k\,v \in \coprod _{k\in \mathbb {Z}_{\ge 0}}\, V^{-k\,{\varvec{m}}}$ (and similarly for $w\in W^{-k\,{\varvec{m}}}$), where the power in $c^k$ depends on the term in the coproduct. Hence, by ${F_{{\varvec{m}}}}^{{\varvec{0}}}$-linearity of $\eta _{X_{F_{{\varvec{m}}}}}$ (which in particular implies $\mathbb {K}$-linearity), we find that $\eta _{X_{F_{{\varvec{m}}}}}$ decomposes into $\mathbb {K}$-linear maps

9.20 $$\begin{aligned} L_{{\varvec{m}}, k}{:}\, V^{-k\,{\varvec{m}}} \longrightarrow W^{-k\,{\varvec{m}}}. \end{aligned}$$

It remains to show that $L_{{\varvec{m}}, k} = L_{k\,{\varvec{m}},1}$, for all ${\varvec{m}}\in \mathbb {Z}^n$ and all $k\in \mathbb {Z}_{\ge 0}$. Consider the ${^{H}}\mathscr {A}_{\mathrm {fp}}$-morphism $f^*{:}\,F_{k\,{\varvec{m}}}\rightarrow F_{{\varvec{m}}}$ defined by $x\mapsto x^k$. The corresponding commutative diagram (9.18) then relates $\eta _{X_{F_{k\,{\varvec{m}}}}}$ to $\eta _{X_{F_{{\varvec{m}}}}}$, and we obtain the desired result $L_{{\varvec{m}}, k} = L_{k\,{\varvec{m}},1}$. This defines a unique ${^{H}}\mathscr {M}$-morphism

9.21 $$\begin{aligned} L:= \coprod _{{\varvec{m}}\in \mathbb {Z}^n}\, L_{{\varvec{m}},1}{:}\,\coprod _{{\varvec{m}}\in \mathbb {Z}^n}\, V^{-{\varvec{m}}} \longrightarrow \coprod _{{\varvec{m}}\in \mathbb {Z}^n}\, W^{-{\varvec{m}}} \end{aligned}$$

and hence by the assumption that *V* and *W* are decomposable also a unique ${^{H}}\mathscr {M}$-morphism $L{:}\,V\rightarrow W$. $\square$

## References

[1] Aschieri, P., Bieliavsky, P., Pagani, C., Schenkel, A.: Noncommutative Principal Bundles Through Twist Deformation. arXiv:1604.03542 [math.QA]

[2] Aschieri P, Dimitrijevic M, Meyer F, Wess J (2006). Noncommutative geometry and gravity. Class. Quantum Gravity.

[3] Aschieri, P., Dimitrijevic, M., Kulish, P., Lizzi, F., Wess, J.: Noncommutative Spacetimes: Symmetries in Noncommutative Geometry and Field Theory, Lecture Notes Physics, vol. 774, p. 1 (2009)

[4] Aschieri, P., Schenkel, A.: Noncommutative connections on bimodules and Drinfeld twist deformation. Adv. Theor. Math. Phys. **18**, 513 (2014). arXiv:1210.0241 [math.QA]

[5] Barnes, G.E., Schenkel, A., Szabo, R.J.: Nonassociative geometry in quasi-Hopf representation categories I: Bimodules and their internal homomorphisms. J. Geom. Phys. **89**, 111 (2015). arXiv:1409.6331 [math.QA]

[6] Barnes, G.E., Schenkel, A., Szabo, R.J.: Nonassociative geometry in quasi-Hopf representation categories II: Connections and curvature. J. Geom. Phys. **106**, 234 (2016). arXiv:1507.02792 [math.QA]

[7] Brain, S., Landi, G.: Moduli spaces of noncommutative instantons: gauging away noncommutative parameters. Q. J. Math. **63**, 41 (2012). arXiv:0909.4402 [math.QA]

[8] Brain, S., Landi, G., van Suijlekom, W.D.: Moduli spaces of instantons on toric noncommutative manifolds. Adv. Theor. Math. Phys. **17**, 1129 (2013). arXiv:1204.2148 [math-ph]

[9] Brzeziński, T., Janelidze, G., Maszczyk, T.: Galois structures. In: Hajac, P.M. (ed.) Lecture Notes on Noncommutative Geometry and Quantum Groups. http://www.mimuw.edu.pl/~pwit/toknotes/toknotes.pdf

[10] Brzeziński, T., Majid, S.: Quantum group gauge theory onquantum spaces. Commun. Math. Phys. **157**, 591 (1993) [Erratum: Commun. Math. Phys. **167**, 235 (1995)] arXiv:hep-th/9208007

[11] Cirio LS, Landi G, Szabo RJ (2011). Algebraic deformations of toric varieties. II: Noncommutative instantons. Adv. Theor. Math. Phys..

[12] Cirio, L.S., Landi, G., Szabo, R.J.: Algebraic deformations of toric varieties. I: general constructions. Adv. Math. **246**, 33 (2013). arXiv:1001.1242 [math.QA]

[13] Cirio LS, Landi G, Szabo RJ (2014). Instantons and vortices on noncommutative toric varieties. Rev. Math. Phys..

[14] Connes, A., Dubois-Violette, M.: Noncommutative finite-dimensional manifolds: spherical manifolds and related examples. Commun. Math. Phys. **230**, 539 (2002). arXiv:math.QA/0107070

[15] Connes, A., Landi, G.: Noncommutative manifolds: The instanton algebra and isospectral deformations. Commun. Math. Phys. **221**, 141 (2001). arXiv:math.QA/0011194

[16] Dabrowski L, Krajewski T, Landi G (2000). Some properties of nonlinear sigma-models in noncommutative geometry. Int. J. Mod. Phys. B.

[17] Dabrowski, L., Krajewski, T., Landi, G.: Nonlinear sigma-models in noncommutative geometry: fields with values in finite spaces. Mod. Phys. Lett. A **18**, 2371 (2003). arXiv:math.QA/0309143

[18] Dabrowski, L., Landi, G., Luef, F.: Sigma-model solitons on noncommutative spaces. Lett. Math. Phys. **105**, 1663 (2015). arXiv:1501.02331 [math-ph]

[19] Kock, A.: Synthetic Differential Geometry, London Mathematical Society Lecture Notes Series, vol. 333. Cambridge University Press, Cambridge (2006)

[20] Landi, G., van Suijlekom, W.: Principal fibrations from noncommutative spheres. Commun. Math. Phys. **260**, 203 (2005). arXiv:math.QA/0410077

[21] Lavendhomme, R.: Basic Concepts of Synthetic Differential Geometry, Kluwer Texts in the Mathematical Sciences, vol. 13. Kluwer, Dordrecht (1996)

[22] Mac Lane S, Moerdijk I (1994). Sheaves in Geometry and Logic: A First Introduction to Topos Theory.

[23] Majid S (1995). Foundations of Quantum Group Theory.

[24] Mathai V, Rosenberg JM (2011). A noncommutative sigma-model. J. Noncommut. Geom..

[25] Mathai V, Sati H (2015). Higher abelian gauge theory associated to gerbes on noncommutative deformed M5-branes and S-duality. J. Geom. Phys..

[26] Moerdijk I, Reyes GE (1991). Models for Smooth Infinitesimal Analysis.

[27] Rieffel MA (1988). Projective modules over higher-dimensional noncommutative tori. Can. J. Math..

